# Advancements in Ocular Therapy: A Review of Emerging Drug Delivery Approaches and Pharmaceutical Technologies

**DOI:** 10.3390/pharmaceutics16101325

**Published:** 2024-10-12

**Authors:** Bhupendra Raj Giri, Deeksha Jakka, Michael A. Sandoval, Vineet R. Kulkarni, Quanying Bao

**Affiliations:** 1 Division of Molecular Pharmaceutics and Drug Delivery, College of Pharmacy, The University of Texas at Austin, Austin, TX 78712, USA; bhupendra@utexas.edu (B.R.G.); sandov@utexas.edu (M.A.S.); vineetkulkarni@utexas.edu (V.R.K.); 2 School of Pharmacy, The University of Mississippi, University, MS 38677, USA; djakka@go.olemiss.edu; 3 Synthetic Product Development, Alexion, AstraZeneca Rare Disease, 101 College Street, New Haven, CT 06510, USA

**Keywords:** ocular therapy, physiological barriers, drug delivery, 3D printing (3DP), hot-melt extrusion (HME)

## Abstract

Eye disorders affect a substantial portion of the global population, yet the availability of efficacious ophthalmic drug products remains limited. This can be partly ascribed to a number of factors: (1) inadequate understanding of physiological barriers, treatment strategies, drug and polymer properties, and delivery systems; (2) challenges in effectively delivering drugs to the anterior and posterior segments of the eye due to anatomical and physiological constraints; and (3) manufacturing and regulatory hurdles in ocular drug product development. The present review discusses innovative ocular delivery and treatments, encompassing implants, liposomes, nanoparticles, nanomicelles, microparticles, iontophoresis, in situ gels, contact lenses, microneedles, hydrogels, bispecific antibodies, and gene delivery strategies. Furthermore, this review also introduces advanced manufacturing technologies such as 3D printing and hot-melt extrusion (HME), aimed at improving bioavailability, reducing therapeutic dosages and side effects, facilitating the design of personalized ophthalmic dosage forms, as well as enhancing patient compliance. This comprehensive review lastly offers insights into digital healthcare, market trends, and industry and regulatory perspectives pertaining to ocular product development.

## 1. Introduction

According to the World Health Organization’s (WHO) 2023 data on blindness and visual impairment, at least 2.2 billion people worldwide suffer from near or distant vision impairment, with approximately half of these instances being untreated or potentially preventable [1]. This widespread health issue carries substantial economic burden, with an estimated USD 411 billion in lost productivity annually on a global basis [2]. In the United States (US), the prevalence of ocular issues is particularly concerning. According to recent data, approximately 90 million Americans aged 40 and above—representing more than 60% (three out of every five) in this demographic—experience visual impairment or ocular disorders [2].

The etiology of vision loss is multifactorial, with the primary conditions contributing to this pervasive issue including, but not limited to, age-related macular degeneration (AMD), glaucoma, cataracts, diabetic retinopathy, and retinal vein occlusion [3,4]. Without proper treatment, these disorders can potentially lead to total blindness. While the pharmaceutical industry has made significant strides in developing various preventative, treatment, and rehabilitation measures that have augmented the effectiveness of approved interventions for eye disorders, these measures are rarely sufficient to eradicate these conditions completely.

The crux of successful treatment lies in the delivery of an effective concentration of a drug to traverse various ocular barriers and reside in the eye for an intended period of time. To date, most of the ocular drug products are applied topically, such as drops, ophthalmic suspensions, emulsions, and ointments. However, these topical formulations suffer from inadequate bioavailability due to precorneal loss factors such as tears and blinking, nonproductive absorption through the conjunctiva, nasolacrimal drainage, and low corneal membrane permeability [5]. As a consequence, the drug may remain in the tear film for a shorter period (approximately 1–3 min) and has poor bioavailability (<5%) [6,7]. These pharmacokinetic shortcomings result in these formulations being unable to reach the disease microenvironment within the eye at a sufficient concentration, thereby resulting in poor efficacy.

Diseases affecting the anterior segment of the eye can be treated with topical formulations. However, there exists a substantial unmet medical need for treatments targeting the posterior segment, which is a significant therapeutic target. Posterior eye diseases are the primary cause of vision impairment in industrialized nations [8]. Despite the challenges associated with treating these conditions, numerous breakthroughs have been made in addressing diseases of the posterior segment. Despite progress in addressing these challenges, such as the growing use of the intravitreal (IVI) route and the approval of several ophthalmic drug products (including implants, solutions, and injectable suspensions) by the US Food and Drug Administration (US FDA), the overall number of ophthalmic drug products remains limited compared to other routes of administration, such as oral drug products. Given this disparity, it is imperative to explore emerging ocular delivery strategies and technologies that show promise for meaningful outcomes in the clinic. Furthermore, advanced technologies require a critical examination for the successful development and manufacture of these emerging ophthalmic formulations. This approach will address the current limitations in treating posterior segment eye diseases and potentially expand the range of available ophthalmic medications.

This review is intended to provide an updated understanding of the anatomical and physiological barriers that impede ocular drug delivery and explore potential strategies for overcoming these challenges. Importantly, it elucidates various ocular diseases and examines emerging drug delivery approaches including implants, liposomes, nanoparticles, nanomicelles, microparticles, iontophoresis, in situ gels, contact lenses, microneedles, hydrogels, bispecific antibodies, and gene delivery. Furthermore, this review stands out by highlighting advanced manufacturing technologies such as 3D printing (3DP), hot-melt extrusion (HME), and injection molding that can enable the fabrication of personalized dosage forms. It covers advantages and limitations alongside case studies associated with these technologies. Lastly, this review examines the role of digital healthcare, current market trends, and industry and regulatory perspectives, providing a holistic view of the current state and future directions in ocular drug delivery.

## 2. Barriers to Ocular Drug Delivery

**Figure 1:**
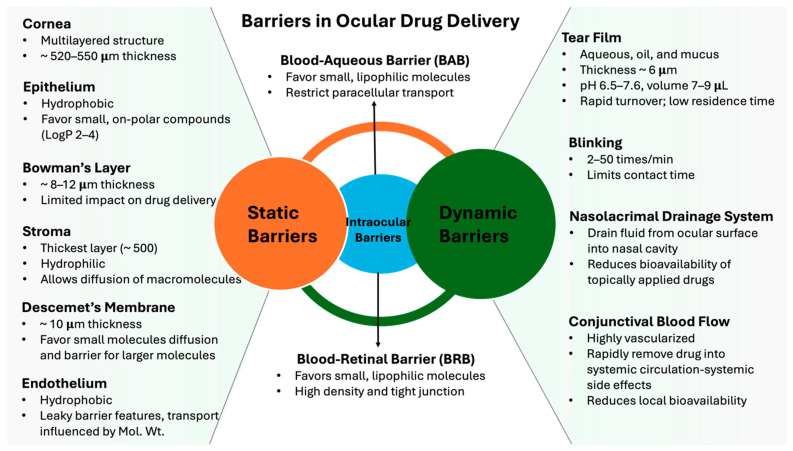
A summary of the key barriers and their properties impeding ocular drug delivery.

The eye, the organ responsible for sight, is one of the most intricate and immune-privileged structures in the body. Its complexity arises from a sophisticated network of specialized tissues, muscles, nerves, glands, and vascularization [9,10]. From a drug delivery perspective, these unique anatomical and physiological features, while essential to ocular health, present both significant challenges and opportunities in the development of efficacious treatments for various eye diseases. While comprehensive overviews of ocular anatomy and physiology are readily available in the literature [9,11,12], this section aims to provide a more focused review of the anatomical and physiological barriers that have been demonstrated to impede ocular drug delivery, and to imply potential strategies for overcoming these obstacles. Understanding these relationships is important in the development and delivery of topical, subconjunctival, intravitreal (IVI), and subretinal therapeutics. To provide better appreciation and address the multifaceted challenges in ocular drug delivery, it is important to understand two principal categories of ocular barriers: static and dynamic (illustrated in Figure 1). These barriers, through their inherent physical properties and physiological processes, exert a significant influence on drug stability, distribution, and ultimately efficacy.

### 2.1. Static Barriers

It is generally agreed that static barriers in the eye are physical attributes that impede the permeation and distribution of drugs (or formulations) due to intrinsic properties and/or composition. The cornea and blood–ocular barriers are two major static barriers that are drug delivery barriers that pharmaceutical scientists should consider during ophthalmic drug development.

The cornea, the transparent front portion of the eye, is the first and primary static barrier to topical ocular drug delivery [13,14]. It comprises distinct hydrophobic and hydrophilic layers, each presenting unique challenges for drug permeation. The outermost layer, the corneal epithelium, is lipophilic and preferentially allows passage of small, non-polar compounds with a LogP between 2 and 4 [15,16]. Tight junctions present between epithelial cells of the cornea are known to restrict the passage of polar compounds [6,17]. As a result, the physicochemical properties of a drug and its dissolution from the formulation are important considerations.

Immediately posterior to the corneal epithelium is the Bowman’s layer, an acellular structure (8 to 12 μm) with limited impact on drug delivery [10,18,19]. The structure–function relationship of the Bowman’s layer has been a subject of extensive debate in the literature. While it was initially believed to serve as a protective barrier against large proteins and to contribute to maintaining corneal shape and curvature [20], these hypotheses have been challenged by others. A comprehensive review by Steven Wilson at the Cleveland Clinic offers an extensive overview and critical examination of past and current beliefs regarding its structure and function, including its role in the movement of macromolecules [18,21]. In the context of drug delivery, the Bowman’s layer is generally considered to have a limited impact. However, this may change due to recent findings, as the Bowman’s layer has been observed to decrease in thickness with age, potentially reducing by up to one-third between ages 20 and 80, and has limited capacity to regenerate, which may warrant careful attention when designing penetrative drug formulations [22].

The stroma, a hydrophilic layer located in the middle of the cornea and posterior to the Bowman’s layer, constitutes about 90% of the corneal thickness (up to 500 μm thick) [19,23]. The stroma is often described in the literature as exhibiting “fluid-like” characteristics with a viscosity of approximately 1.5-fold that of water [24]. For polar compounds, the stroma generally does not present a rate-limiting barrier. Maurice and Mishima [25] demonstrated that molecules with a molecular weight of up to 500 kDa can diffuse across the stromal tissue. However, for non-polar compounds, the stroma has been shown to impart significant barrier properties [26]. Furthermore, the stroma also exhibits a “reservoir effect” for drugs that successfully penetrate through the epithelium. It is worth noting that this phenomenon has significant implications [5,27]. While potentially advantageous for polar drugs or when targeting the stroma itself, this reservoir effect may be counter-effective for achieving therapeutic concentrations in the posterior segments of the eye [28].

At the interface between the stroma and endothelium lies the Descemet’s Membrane [29]. While the Descemet’s Membrane does not significantly restrict the diffusion of small molecules, it does function as a barrier for larger macromolecules and particles. This selective permeability is particularly relevant for drugs dosed directly into the stroma, as they may encounter challenges in penetrating the endothelium [30].

The innermost layer of the cornea is the corneal endothelium, which functions as the second lipophilic barrier. The corneal endothelium is responsible for regulating the hydration of the cornea and for maintaining corneal clarity and its function. The cells that make up the corneal endothelial cells are characterized as comprising a high density of Na+/K+ ATPase pumps on their basolateral membrane, actively pumping ions from the stroma to the aqueous humor [31]. Additionally, bicarbonate transporters [32], aquaporin [33], water channels, and tight junctions create osmotic gradients to regulate the movement of fluids across the cell membrane. While the corneal endothelium is often classified as a lipophilic barrier and described as ‘more permeable than the corneal epithelium’ due to its leaky barrier features and directional transport, it is important to note that the movement of drugs across the endothelium is influenced by drug molecular weight [34]. This characteristic is important when considering the size of drugs intended to be delivered.

Beyond the cornea, the eye possesses two sophisticated barrier systems that regulate the exchange of substances between the bloodstream and ocular tissues: the blood–aqueous barrier and the blood–retinal barrier. These sophisticated barrier systems are well known to impart significant challenges for systemically administered drugs to reach therapeutic concentrations within the eye. The blood–aqueous barrier, formed by the non-pigmented epithelium of the ciliary body and iris blood vessel endothelium, regulates the composition of the aqueous humor [35]. The blood–retinal barrier, made up of RPE and retinal blood vessel endothelial cells, functions to protect the neural retina tissue [36,37].

From a drug delivery perspective, understanding types of static barriers and their ability to limit or allow the passage of compounds is an important consideration when developing effective drug delivery formulations for the eye. Strategies for overcoming static barriers are discussed later in this review.

### 2.2. Dynamic Barriers

While static barriers form the structural foundation of eye defense mechanisms, dynamic barriers involve various physiological processes that can actively remove or dilute drugs from the eye. This section will discuss the principal dynamic barriers including the tear film, blinking, nasolacrimal drainage, and conjunctival blood flow.

The tear film represents a significant dynamic barrier in ocular drug delivery. First, it is approximately 6 μm thick, serves to maintain ocular comfort through lubrication, and ensures clear vision by creating a smooth optical surface over the cornea, contributing substantially to the eye’s refractive power. While the composition of the tear comprises three layers (oil, water, and mucus), it exhibits dynamic properties based upon the stimulus for tear production. For instance, tears produced in response to emotional stimuli differ chemically from those triggered by irritants. This compositional variability introduces another layer of complexity to ocular drug delivery strategies.

The pH of the tear film has been reported to range from 6.5 to 7.6, and attention needs to be paid to maintaining drug solubility and avoiding irritation during formulation development [38]. Moreover, the presence of various antimicrobial components such as lysozyme, lactoferrin, and immunoglobulin A in tears, while providing natural defense mechanisms, may also interact with topically applied drugs, further complicating delivery strategies [11].

Another important aspect of the tear film’s dynamic nature is its rapid turnover rate. With a flow rate of approximately 1 μL/min and a total volume of only 7 to 9 μL, the entire tear film undergoes replacement in a matter of minutes [39]. This process is regulated by the lacrimal functional unit, which is a sophisticated system comprising ocular surface nerves, the central nervous system, and ocular glands [40]. It has been reported that the human eye blinks 2 to 50 times per minute [41], further contributing to this frequent exchange. While essential for maintaining ocular health, this rapid turnover has been shown to impart challenges for topical drug delivery by limiting the residence time of drugs on the eye’s surface [42].

The nasolacrimal drainage system also plays an important role in maintaining ocular homeostasis and has been shown to impact the residence time of topically applied drugs. The main purpose of the nasolacrimal drainage system is as it sounds, to remove tear fluid from the ocular surface. This drainage process begins at the small openings located at the medial aspect of both the upper and lower eyelids called puncta. These puncta lead to narrow ducts called canaliculi, which converge to form the common canaliculus before emptying into the lacrimal sac. Subsequently, fluid drains into the nasolacrimal duct, ultimately opening into the inferior meatus of the nasal cavity [43]. Since this nasolacrimal drainage system is continuous and important for maintaining healthy tear volume, it does pose significant challenges for ocular delivery. It has been estimated that up to 80% of an eye drop can be lost through nasolacrimal drainage within 15–30 s of application [44,45]. This rapid clearance can substantially reduce the bioavailability of topically applied medications and necessitates frequent dosing to maintain therapeutic levels. The rate of nasolacrimal drainage is influenced by various factors, including tear production rate, blinking frequency, and the volume of topically applied drops [46]. Larger drop volumes, ironically, can lead to increased drug loss through overflow and stimulation of reflex tearing [47,48]. Furthermore, drugs that enter the nasolacrimal system may be absorbed through the nasal mucosa, potentially leading to systemic exposure and unintended side effects [49]. This phenomenon is particularly relevant for drugs with high mucosal permeability or those targeting conditions where systemic exposure is undesirable. Several previous reviews have educated the dynamics of the nasolacrimal drainage system and proposed formulation approaches to either increase residence time through viscosity enhancers or physically block puncta to temporarily suspend drainage and achieve therapeutic concentrations [47,50,51,52].

The final dynamic barrier of note is the conjunctiva and its associated blood flow. The conjunctiva, a thin, transparent-pinkish, mucous membrane covering the anterior eye and inner surface of the eyelids, is made up of epithelial cells and connective tissue that is rich is blood vessels [53]. The vascular system of the conjunctiva is made up of arterioles, capillaries, and venules that permeate the substantia propria and is collectively referred to as the conjunctival blood flow [53]. This extensive vascularization gives the conjunctiva not only its distinct pink color but also unique attributes that can impact drug delivery. For example, drugs that happen to penetrate the conjunctival epithelium will likely encounter rapid removal into the systemic circulation, thereby compromising local bioavailability and potentially leading to unwanted systemic effects.

These dynamic barriers, when considered collectively, represent challenges to ocular drug delivery. Their combined barrier effects often require the development of innovative formulation and device strategies to overcome their effects and achieve efficacious drug concentrations within the target eye tissue. The complexity of these barriers highlights the need for multiple drug delivery strategies to consider both drug physicochemical properties and physical processes that govern drug disposition into the eye. Several recent reviews offering more in-depth analysis and specific examinations of the latest advancements in the understanding of ocular physiology are available [5,28,54].

## 3. Common Eye Diseases and Treatment

This section of this review aims to provide a high-level overview of ocular diseases that have garnered significant attention in drug development. While this section is intended to provide a concise refresher on disease states, we strongly encourage readers to peruse in-depth literature reviews for each disease to gain a more appreciable understanding and deeper insight into its etiology, pathophysiology, symptomatology, and historical context.

### 3.1. Glaucoma

Glaucoma, a leading cause of permanent blindness, is characterized by retinal ganglion cell degeneration and optic nerve damage [55]. This disease affects approximately 80 million people globally, with elevated intraocular pressure (IOP) as the primary modifiable risk factor [56,57].

Glaucoma can be classified into four distinct categories based on specific anatomical features of the eye and underlying mechanisms: primary open-angle glaucoma (POAG), angle-closure glaucoma (ACG), normal-tension glaucoma (NTG), and secondary glaucoma. Among these types of glaucoma, POAG is the most common form; in it, the aqueous humor outflow is compromised due to the reduced permeability of trabecular meshwork, thereby increasing IOP [58]. ACG is the second most common form of glaucoma and involves iris bulging that narrows the anterior chamber angle of the iris and obstructs aqueous humor outflow [59].

Treatment strategies are largely focused on reducing IOP and preserving the optic nerve. First-line therapy generally typically involves topical medications such as prostaglandin analogs, beta-blockers, alpha-agonists, and carbonic anhydrase inhibitors [60,61]. These drugs have been shown to work through various mechanisms to decrease aqueous humor production or increase outflow. For patients who are unresponsive to these treatments, laser treatments such as selective laser trabeculoplasty (SLT) or argon laser trabeculoplasty (ALT) have been shown to enhance aqueous outflow [62]. Surgical interventions, including trabeculectomy and drainage implant surgery, create alternative outflow pathways for refractory glaucoma cases [63]. More recently, less invasive procedures such as microinvasive glaucoma surgery (MIGS) offer additional treatment options with potentially lower risk [64]. The choice of a specific treatment strategy is generally contingent upon several factors, mostly glaucoma type, severity, and individual patient factors, emphasizing the need for targeted and more effective treatment in the management of glaucoma.

### 3.2. Dry Eye Disease/Dry Eye Syndrome

Dry eye disease (DED) is a complex and multifaceted disorder affecting at least 16 million Americans. It is characterized by poor tear quality and inadequate lubrication of the ocular surface, which often leads to discomfort and disturbances in seeing [65]. DED is commonly classified into two main categories: aqueous-deficient dry eye (ADDE) and evaporative dry eye (EDE), though patients often experience a combination of both [66].

As previously discussed, the tear film is an important component of ocular health and is made up of lipids, aqueous, and mucin layers. In DED, this tear composition is compromised, resulting in the instability of the tear film and an increase in osmolarity [39,67]. These changes trigger inflammatory responses, producing pro-inflammatory cytokines and enzymes that further damage the ocular surface and tear-producing glands, creating a “self-perpetuating cycle” of tear instability, hyperosmolarity, and inflammation, and as a result, damage glands [68,69].

Treatment strategies generally aim to restore the surface of the eye and minimize symptoms. Non-pharmacological interventions often include environmental modifications and lifestyle changes [70]. Artificial tears and lubricating drops are commonly used for symptomatic relief. For moderate to severe cases, prescriptions such as cyclosporine and lifitegrast are used to target underlying inflammation [71]. Short-term corticosteroid eye drops are often prescribed to reduce flare-ups. Advanced treatments, including punctal plugs, scleral contact lenses, and intense pulsed light therapy, are also available and used for severe cases [72].

### 3.3. Diabetic Retinopathy and Diabetic Macular Edema

Diabetic retinopathy (DR) and diabetic macular edema (DME) are serious microvascular complications of diabetes affecting the retina. DR is characterized by progressive damage to retinal blood vessels, while DME is characterized by the accumulation of fluid in the macula [73].

The pathophysiology is generally believed to be attributed to chronic hyperglycemia, including increased oxidative stress and the formation of advanced glycation end-products in the retina [74,75]. These changes lead to a cascade of events affecting the vascularization of the retina, such as the loss of pericytes, thickening of the basement membrane, and increased permeability of the blood–retinal barrier that results in the leakage of fluids, proteins, and lipids from blood vessels into retinal tissue contributing to edema [76,77,78]. Consequently, these vascular changes lead to retinal ischemia as the compromised blood vessels fail to deliver adequate oxygen and nutrients to retinal cells. This ischemia further induces compensatory mechanisms such as the production of vascular endothelial growth factor (VEGF) to promote the growth of new blood vessels that further increase vascular permeability [79].

Treatment of DR and DME generally aims to minimize disease progression and loss of vision. Glycemic control and management of major risk factors such as hypertension are priorities [80]. Advanced forms of DR are often managed with panretinal photocoagulation (PRP). More recently, intravitreal anti-vascular endothelial growth factor (anti-VEGF) injection has become the standard of care with ranibizumab, aflibercept, and off-label bevacizumab [81,82,83]. In advanced cases, vitreoretinal surgery is often necessary to address complications like vitreous hemorrhage or tractional retinal detachment [80].

### 3.4. Retinal Vein Occlusion

Retinal vein occlusion (RVO) is a significant vascular disorder of the retina and is the second most common cause of vision loss due to obstruction of retinal venous circulation [84], affecting more than 16 million people in the US [85]. RVO is classified into two main types differing in the location and extent of venous obstruction: central retinal vein occlusion (CRVO) and branch retinal vein occlusion (BRVO). CRVO affects the central retinal vein, while BRVO affects the branched retinal vein [86]. Both types can lead to sudden vision loss due to obstructed blood flow, resulting in retinal ischemia, bleeding, fluid leakage, and retinal swelling or macular edema [87].

Treatment strategies for RVO are focused on managing major complications and preventing vision loss, with treatment based on RVO type. For macular edema secondary to RVO, intravitreal anti-vascular endothelial growth factor (anti-VEGF) agents are the first line [88]. Similar to DR and DME, intravitreal corticosteroids such as dexamethasone implants are also used, more often in situations where DR/DME is refractory to anti-VEGF treatment.

### 3.5. Uveitis

Uveitis is an inflammatory condition that affects the uveal tract or middle layer of the eye comprising the iris, ciliary body, and choroid [89]. If left untreated, uveitis has the potential to lead to permanent vision loss, with approximately 30,000 new cases of blindness reported annually in the US [90].

Clinically, uveitis is classified into four types based on the primary type of inflammation: anterior, intermediate, posterior, and panuveitis. Its pathogenesis is generally influenced by multiple factors including infections, autoimmune processes, and trauma [89]. The inflammatory condition often leads to increased uveal blood vessel permeability, altered aqueous humor composition, and inflammatory cell influx into ocular tissues [91,92]. These changes can result in physiological disruptions such as IOP and disruptions in the blood–retinal barrier [93,94].

Treatment of uveitis is primarily intended to reduce inflammation and prevent further complications. Topical or intravitreal corticosteroids are the standard of care for anterior uveitis and intermediate or posterior uveitis, respectively [95]. Systemic corticosteroids are generally reserved for bilateral or severe cases. Non-steroid treatment such as antimetabolites (e.g., methotrexate, mycophenolate), calcineurin inhibitors (e.g., cyclosporine, tacrolimus), and monoclonal antibodies (e.g., adalimumab, infliximab) are available for chronic and recurrent uveitis [96].

### 3.6. Age-Related Macular Degeneration

In individuals over 50 years old, age-related macular degeneration (AMD) is the leading cause of vision loss, affecting over 18 million people in the US [97]. AMD is projected to affect 288 million globally by 2040 [98]. This degeneration primarily affects the macula but also affects other ocular tissues such as the light-sensitive cells in the retina, retinal pigment epithelium (RPE), Bruch’s membrane, and the vascular layer supplying blood to the outer retina or choroid [99]. The pathogenesis of AMD is complex and not entirely understood, with major risk factors including age-related changes in the immune system, oxidative stress, and lipid metabolism [100].

AMD can be classified into two types: dry (90% of cases) and wet forms. Dry AMD is generally characterized by drusen deposits beneath the retina and loss of RPE cells, photoreceptors, and choriocapillaris [101]. Wet AMD involves abnormal blood vessel growth beneath the retina, leading to more severe vision loss [102].

Treatment of dry AMD is focused on lifestyle and nutritional supplements, including omega-3 fatty acids and zinc (age-related eye disease study (AREDS) formulation) [103]. Wet AMD treatment generally requires intravitreal anti-VEGF injections to reduce choroidal neovascularization. Combination therapies like anti-VEGF and photodynamic therapy are also available and are becoming more popular [104].

## 4. Emerging Ocular Drug Delivery Systems

### 4.1. Ocular Implants

Ocular implants represent a significant advancement in ophthalmic drug delivery, offering numerous advantages over conventional administration methods. These are pharmaceutically developed to deliver drugs to a predetermined ocular location within a specific timeframe, thereby circumventing the need for multiple injections into the ocular tissues that could potentially exacerbate infections and inflammatory processes [105]. The primary benefits of ocular implants over more conventional techniques for administering drugs to the eye include localized delivery and providing consistent therapeutic drug concentrations directly at the site of action [106].

The installation of implants varies depending on the targeted region of the eye. A subconjunctival implant is generally employed for anterior segment disorders, while intravitreal (IVI) and suprachoroidal procedures are utilized for posterior segment diseases. Intrascleral implants represent a versatile option for both anterior and posterior segment conditions. Regardless of the implantation site, there is a considerable likelihood of drug diffusion to both portions. As expected, the implantation process varies depending on the target location. Subconjunctival implants are applied to the sclera through a tiny incision in the conjunctiva. Intrascleral devices, which are implanted in a small scleral pocket at half the total scleral thickness, are beneficial for treating posterior segment diseases with reduced systemic drug absorption compared to subconjunctival or peribulbar injections. This is due to their proximity to the site of action relative to conventional transscleral devices. Intravitreal insertion of ocular implants provides drug direct access to the posterior segment’s target tissues. The implantation process involves an applicator, eliminating the need for sutures due to needle administration or a sclerotomy site. The pars plana, which is posterior to the lens and anterior to the retinal insertion, is frequently chosen as the site of implantation due to its reduced risk of harming ocular structures.

Ocular implants can be categorized into two primary types: biodegradable and non-biodegradable based on rate-controlling polymers or systems used in the products. Non-biodegradable implants offer the benefit of providing sustained and controlled drug release over prolonged durations (up to several years); however, they require replacement or removal once the drug is depleted. In contrast, biodegradable implants present several advantages, including flexibility in shaping into various forms, the possibility of implantation outside a hospital setting, the absence of removal procedures, and the ability to extend the drug’s half-life [107]. Collectively, these benefits make biodegradable implants an attractive option in ocular drug delivery.

To date, several ophthalmic implant products have received approval from the US FDA (Table 1). Of these, six implants are non-biodegradable, and two are biodegradable. With the exception of iDose TR and Suvismo, all non-biodegradable ophthalmic implants contain the rate-controlling polymer polyvinyl alcohol (PVA). The two biodegradable implants are composed of the bioabsorbable copolymers poly (D, L-lactide) (PLA, PDLA, PDLLA) and/or poly (lactic-co-glycolic acid) (PLGA). The most recently approved implant, iDose TR, is a travoprost implant measuring 1.8 mm × 0.5 mm. It contains four main components: a drug reservoir, a semipermeable membrane for rate controlling, a scleral anchor, and a titanium cap, which holds the membrane in place. Suvismo (a ranibizumab implant) is the first approved refillable intravitreal implant, and it delivers a ranibizumab injection through a port delivery system (PDS) every 6 months per fill. The PDS for ranibizumab comprises four components contained in silicone encasement: a hollow and refillable drug reservoir, a release-controlling element (titanium medium), a septum, and an extrasclera flange.

Vitrasert (ganciclovir intravitreal implant, now discontinued) is the first FDA-approved non-biodegradable ophthalmic implant with PVA and EVA as controlling polymers, indicated for CMV retinitis in patients with acquired immunodeficiency [109]. The implant releases ganciclovir at a rate of 1 mcg/hour for 5 to 8 months and is positioned at the pars plana, extending into the vitreous cavity. However, while it is effective, its potential complications include endophthalmitis, retinal detachment, and vitreous hemorrhage [110]. In addition, the implant needs to be changed after releasing the drug for a period of five to eight months. Following the approval of Vitrasert, three other PVA-based non-biodegradable implants of fluocinolone acetonide were approved by the US FDA (from 2005 to 2018) for noninfectious uveitis or DME with much longer durations (up to 36 months or 3 years). Attempts to include high-molecular-weight compounds in non-biodegradable implants have not yielded much success [111]. Encapsulated cell technology (ECT) presents a promising cell-based delivery method for administering therapeutic drugs to the eye. In ECT, altered cells are enclosed in a hollow, semipermeable membrane tube that blocks the entry of immune cells while allowing nutrients and healing agents to pass through. The polymer part is sealed at both ends, and the anchoring end is inserted at the pars plana and secured to the sclera with a titanium loop [112].

Biodegradable implants have gained attention in ocular drug delivery due to their ability to achieve steady-state delivery and for being biocompatible. These implants are typically used to treat acute-onset diseases that need a loading dose and subsequent drug tapering over a period of one day to six months. As mentioned above, the most used excipients for biodegradable implants are the copolymers PLA and/or PLGA. The drug release from biodegradable ophthalmic implants typically follows first-order kinetics, with initial burst release followed by rapidly declining concentrations [107]. Another advantage of using biodegradable implants to tailor drug delivery to the course of a disease is that they allow for dose and treatment flexibility, ranging from a couple of weeks to longer durations of months or years, depending on the ratio of PLA to PLGA in the polymer. PLGA has been used extensively in the fabrication of biodegradable implants, extending drug release over weeks to months [113]. These implants can generally accommodate higher drug loading because of their larger size and smaller surface-to-volume ratio [114]. Ozurdex is one example of a biodegradable ocular implant product that delivers dexamethasone. It was approved by the US FDA in 2009 for treating macular edema and contains 0.7 mg of dexamethasone to be delivered to the vitreous in a controlled manner. Utilizing Allergan’s NO-VADUR technology, this rod-shaped (approximately 6.0 mm × 0.46 mm) PLGA-based pellet gradually degrades into lactic and glycolic acid, allowing continuous dexamethasone to be delivered for up to 6 months. The Ozurdex implant is administered through intravitreal injection, representing a significant advancement in the treatment of ocular drug delivery.

Despite the advancements in biodegradable implants, significant challenges persist in their development and commercialization, particularly for macromolecules used in treating eye disorders such as AMD. One of the primary issues associated with PLGA-based implants is the need for invasive surgical procedures to insert the implant into the vitreous or sclera of the diseased eye [115].

### 4.2. Liposomes

Liposomes are small vesicles consisting of one or more concentric lipid bilayers that are separated by aqueous compartments. The classification of liposomes is based on two factors: the number of phospholipid bilayers and their size. Accordingly, they are categorized as multilamellar (more than one bilayer and generally larger than 300 nm), small unilamellar (generally 10 nm to 100 nm), and large unilamellar (generally 100 nm to 300 nm) [116]. The effectiveness of liposomes in ocular drug delivery is attributed to their ability to form a strong bond with the surfaces of the cornea and conjunctiva, thereby enhancing ocular drug absorption [117]. Drugs having low solubility, low partition coefficient, low absorption, or medium to high molecular weights can particularly benefit from this approach. Liposomes’ effectiveness in ocular applications stems from their superior biocompatibility, cell-membrane-like structure, and capacity to encapsulate both hydrophilic and hydrophobic drugs. Numerous studies have demonstrated the effectiveness of liposomes in anterior and posterior segment ocular delivery [118].

**Figure 2:**
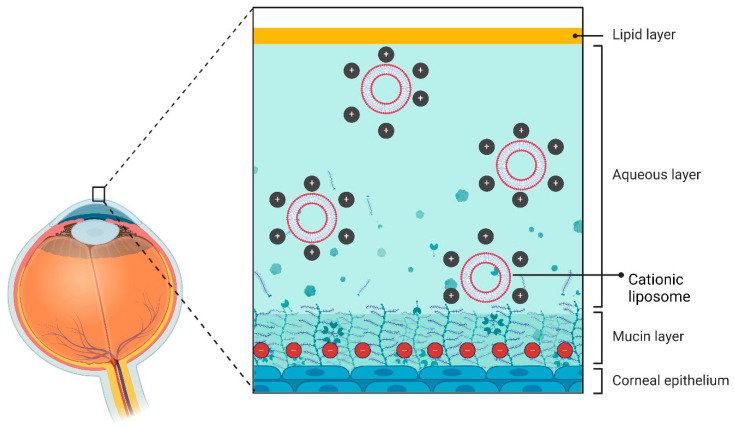
An illustration depicting the interaction of cationic liposomes on the cornea with the negatively charged mucin layer. Created with BioRender.com (accessed on 3 August 2024).

The surface charge of liposomes imparts a significant role in their effectiveness as an eye drug delivery vehicle (Figure 2). Positively charged liposomes exhibit superior absorption at the negatively charged corneal surface compared to neutral or negatively charged liposomes. Lipids used in liposomes are generally both biocompatible and biodegradable, offering site-specific delivery and sustained release properties. However, there are some caveats in liposomal ophthalmic drug delivery. For instance, when phospholipid components of the liposomes are produced in suspension form, they may result in vitreous cloudiness and obscured vision [119]. In addition, studies have shown that liposome half-lives shorten in inflammatory eyes, although the exact cause is still unclear. It could be related to modifications in the blood–retina barrier and infiltration of inflammatory cells, which increase liposome degradation [120].

In recent years, numerous studies have evaluated liposomal systems for topical ophthalmic drug delivery. The development of liposomal fluconazole and a comparison with a commercially available formulation were carried out by Sanap et al. in 2022 [121]. Their results showed that the liposomal fluconazole release kinetics followed a zero-order pattern and that the ex vivo permeation flux of the liposomal fluconazole liposomes was higher than that of the formulation that was sold commercially. Preclinical research revealed no signs of hemolysis or discomfort in the eyes. When the fluconazole liposomes were compared to the commercial formulation, the area under the curve and mean residence time were found to have increased 2.36- and 3.05-fold, respectively [121]. In another work, a hybrid formulation of liposomes and hydroxypropyl methylcellulose (HPMC) was loaded with a poorly water-soluble antiglaucoma drug, acetazolamide. These liposomes were dispersed in a solution including trehalose, borates, and erythritol and demonstrated a 30-fold increase in ocular bioavailability compared to non-liposomal acetazolamide. The improvement was ascribed to two aspects: (1) HPMC increased the formulation viscosity, leading to extended ocular residence time, and (2) the dispersion medium (trehalose, borates, and erythritol) mimicked the tonicity and pH of precorneal film. Additionally, the hybrid system not only enhanced the hypotensive effect of the drug but also maintained the drug’s local effect for a prolonged duration (several hours), potentially reducing the need for frequent administrations and improving patient adherence to treatment [122,123]. The impact of liposome characteristics on retinal penetration has also been investigated. Tavakoli and colleagues assessed the effects of surface charge, coating, and particle size of liposomes on their ability to penetrate the retina using an in vitro bovine explant system. Their findings highlight the significance of particle size, demonstrating that small liposomes (~50 nm) can reach the retina more effectively than larger liposomes (~100 nm). Furthermore, PEGylation and anionic surface charge help retinal liposomes distribute more evenly [124].

To date, only several brand liposomal products (e.g., Doxil, Ambisome, Exparel, Onivyde) have been approved as injectables by the US FDA due to the complexity of liposomal drug product development in general. The US FDA released guidance for the industry, “*Liposome Drug Products Chemistry, Manufacturing, and Controls; Human Pharmacokinetics and Bioavailability; and Labeling Documentation*”, in 2015 and revised it in 2018. Benefiting from the guidance, approximately 15 generic liposomal products have been approved by the agency, mostly within five years (after 2020). Only two commercial liposomal products are available for the treatment of eye disorders: Visudyne^®^ (intravenous injectable with systemic exposure) and Tears Again^®^. Visudyne^®^ contains verteporfin, a photosensitizer used in photodynamic therapy to treat pathological myopia, AMD degeneration, and subfoveal choroidal neovascularization [125]. Tears Again^®^ is a phospholipid liposome spray authorized as an eye lubricant for managing dry eye conditions. Clinical trials have demonstrated notable benefits of this liposomal spray compared to triglyceride-containing eye gel and a balanced salt solution [126,127].

### 4.3. Nanoparticles

Polymeric nanoparticles (NPs) are made of several biodegradable polymeric materials and have a diameter of less than 1000 nm [128]. In ocular drug delivery, NP size has a substantial impact on drug loading and delivery. In general, smaller nanoparticles provide greater stability and biodistribution. For topical use, NPs between 200 and 400 nm are best suited for ocular delivery of drugs, as they are readily absorbed by the cornea and conjunctiva. They show decreased eye irritation, enhanced penetration across ocular barriers, and improved mucoadhesion [129]. The surface charge of NPs is important; cationic NPs tend to stay longer on the ocular surface due to their interaction with negatively charged tissues, while anionic NPs have other features [130].

These versatile systems are often developed using a wide range of excipients, including lipids and proteins, and synthetic and natural polymers, such as PLA, PLGA, albumin, sodium alginate, chitosan, and polycaprolactone are commonly used for developing nanoparticles. Nanoparticles are further categorized into two primary types: nanocapsules and nanospheres, which are tiny capsules having a central hollow encircled by a polymeric membrane. The benefits of NPs are twofold: (1) the drug is enclosed within the produced polymeric lattice, and (2) the drug is evenly distributed throughout the polymeric lattice [131]. The application of nanoparticles in ocular drug delivery offers several advantages. Many research groups are investigating nanoparticulated engineered forms to create delivery systems that are specifically designed to transport drugs to the anterior and posterior ocular regions. Their small size of nanoparticles contributes to reduced ocular tissue irritation and enables sustained delivery, potentially eliminating the need for repeat doses. This formulation attribute is particularly beneficial in promoting patient compliance. To further improve the efficacy of topical ocular formulations, mucoadhesive nanoparticles were developed and designed to prolong residence time on the ocular surface. The versatility of nanoparticles is highlighted by various types under investigation.

A unique single-dose nanoparticle/nanofiber system was developed by Khalil et al. to create a unique, efficient substitute for the traditional dosage form in the treatment of ocular infections in order to decrease the frequency of topical azithromycin administration and increase patient adherence. A formulation composed of PLGA and Pluronic F-127^®^ combined to produce azithromycin-loaded polymer nanoparticles was embedded in electrospun poly(N-vinyl pyrrolidone) nanofibers to make a hybrid ocular insert. The multilayered nanofiber/nanoparticle system was mucoadhesive and biodegradable. The hybrid system allowed the drug to be released for more than ten days, offering benefits such as reduced dosing frequency, increased therapeutic efficacy, fewer adverse reactions, and enhanced patient compliance [132].

In another significant study, tacrolimus-loaded polymeric nanoparticles for DED treatment were developed using the ionotropic gelation process with natural polymer gellan gum. This formulation demonstrated prolonged drug release and increased precorneal retention. Importantly, the nanoparticle formulation was found to be non-toxic and safe for use in the eyes based upon various safety tests, including the Draize, chorioallantoic membrane, and Hen’s egg tests, as well as a histological analysis. In a rabbit model, tacrolimus nanoparticles showed efficacy in reducing DED symptoms [133]. Furthermore, Mohammed et al. developed a chitin-based nanoparticle formulation for the ocular delivery of the antifungal drug fluconazole. This formulation exhibited a controlled, two-step release profile, with only 15% of the drug being released in the first 48 h [134]. Overall, the nanoparticulate system aims to maintain delivery for extended periods of time as an alternative to administering drugs to the posterior portion of the eye. As previously mentioned for liposomes, the effectiveness of nanoparticles in targeting the posterior segment of the eye is primarily determined by their size and surface characteristics.

### 4.4. Nanomicelles

Nanomicelles, ranging from 10 to 100 nm in size, are characterized by an internal hydrophobic fatty acyl chain and an outer hydrophilic polar head. This amphiphilic attribute allows nanomicelles to function effectively as surfactants or polymers, enabling them to deliver both water-soluble and/or poorly soluble drugs to the anterior and posterior parts of the eye [135]. Cholkar et al. showed the important boundaries in utilizing these systems to deliver drugs to ocular tissues. The primary advantages of nanomicelles include their ease of preparation, small size, and excellent capacity to encapsulate high amounts of drugs. Consequently, these formulations with such morphology enable the administration of drugs with higher bioavailability and improved therapeutic outcomes. Notably, nanomicellar formulations have also shown promise in gene therapy for the eye. An important factor influencing the transport and absorption of nanomicelles into ocular tissues is the size of the particles. Generally, smaller particle sizes correlate with higher permeability of the nanomicelles, thereby enhancing their ability to penetrate ocular barriers effectively [136,137].

Recent studies have highlighted the potential of nanomicelles in enhancing drug delivery in various animal models. The unique properties of nanomicelles, including their ultrasmall particle size, amphipathic nature, and high drug encapsulation efficiency, make them particularly effective for ocular applications [138]. Studies have demonstrated that nanomicelles can significantly improve the solubility and bioavailability of poorly water-soluble drugs. A notable example is the work performed by Ping Lu et al. They developed nanomicelles containing Butenafine (BTF) using the copolymer of TPGS to treat fungal keratitis (FK). This formulation demonstrated improvements in drug solubility and corneal permeability. In particular, the BTF-loaded TPGS nanomicelle formulation increased the solubility of BTF by 6000 times and enhanced its permeability through the corneal barriers. In vivo studies on FK mouse models have shown that BTF-NM formulations markedly improved the therapeutic outcomes against FK compared to conventional treatments. This enhanced efficacy was attributed to several factors such as the ability of nanomicelles to maintain a prolonged residence time on the ocular surface, facilitate drug penetration through the corneal epithelium via endocytosis, and achieve higher drug concentrations at the site of infection [139]. These findings suggest that nanomicelle-based drug delivery systems could offer a significant advancement in the treatment of various ocular diseases. By overcoming the inherent barriers to drug absorption in the eye, nanomicelles have the potential to improve ocular drug delivery.

### 4.5. Microparticles

Microparticles are also a significant advancement in ocular drug delivery as they have been shown to offer sustained release and improved bioavailability. These microscopic polymeric particles, suspended in a liquid medium, can carry drugs that are either covalently bonded to the polymer backbone or physically distributed throughout the matrix [140]. Microparticles vary in size from 1 to 1000 µm and are categorized into two types: microspheres and microcapsules [141]. The effectiveness of microparticles is due to their ability to settle in the ocular cul-de-sac following topical application. This positioning allows for drug release via chemical reaction, diffusion, and/or polymer breakdown. Precorneal residence time is extended by microparticles, resulting in a sustained and continuous release of the drug. This results in decreased dose frequency and increased drug ocular bioavailability. However, the relatively large particle size has been shown to cause irritation to the eyes. Ideal polymers for ophthalmic microparticles should exhibit biodegradation, adhesion, and biocompatibility properties. Microparticles can usually only be injected into the eye in small volumes (50 µL to 100 µL) [142].

Naguib et al. developed PLGA microparticles loaded with EPO-R76E, a mutant version of EPO, showing protective effects on retinal neurons and optic nerve axons without causing a large increase in hematocrit. Glaucoma animals that received an intravitreal injection of PLGA.EPO-E76E showed neuroprotective action, inhibited increases in retinal O_2_ levels, and the activation of the NRF2/ARE antioxidant system [143]. Another study investigated the use of a unique intraocular delivery method using PLGA microspheres loaded with three neuroprotective drugs (DEX, melatonin, and coenzyme Q10) for the treatment of glaucoma. A mouse model of chronic ocular hypertension was used to evaluate single intravitreal injections of microspheres co-laden with three drugs compared to those loaded with only one drug or empty ones. After 21 days, the group receiving treatment with the devices coloaded with the three drugs showed signs of neuroprotection [144]. In order to reduce inflammation caused by intravitreal injection of lipopolysaccharide (LPS) into rabbit eyes, Barcia et al. investigated the efficacy of PLGA microspheres loaded with dexamethasone (DEX). Within 30 days of treatment, eye inflammation was significantly reduced, and the eyes were no longer sensitive to a second LPS test [145].

### 4.6. Iontophoresis

Ocular iontophoresis is gaining popularity for its non-invasive method of drug delivery to both the anterior and posterior segments of the eye. This technique utilizes low electrical currents to transport ionized drugs through membranes by two mechanisms: electro-osmosis and migration [146]. The process involves using an electrode with the same charge as the drug for delivery, while a ground electrode with the opposite charge is placed elsewhere on the body to complete the circuit. The drug acts as the tissue’s conductor of electricity. While human studies are limited, animal experiments have demonstrated the potential of transcorneal and trans-scleral iontophoresis for delivering various ophthalmic drugs, including oligonucleotides [147]. Iontophoresis not only eliminates the hazards associated with intravitreal injections and surgical implantation but also does not extend the half-life of the medicine. Trans-scleral iontophoresis has shown promise in animal experiments for administering anti-VEGF drugs to the retina and choroid. When the current density is adjusted to 6.25 mA/cm^2^ during iontophoresis, the drug load in the cornea can be increased to 120 ng/mg. This accumulation of the drug in the cornea can facilitate a slow, continuous release into the aqueous humor, creating an in situ drug depot for prolonged therapeutic effect [148].

Three types of ocular iontophoresis have been developed: transcorneal, trans-scleral, and corneoscleral. The sclera’s permeability to high-molecular-weight compounds and its large surface area make it suitable for drug delivery to the posterior segment. It also has a higher degree of moisture and fewer cells [149]. Drug transfer to the posterior region is made possible via transscleral administration. Importantly, it is a simple and non-invasive procedure [150]. Iontophoresis offers several benefits over traditional ocular delivery methods including needles and topicals. When compared to topical eye drops, it can achieve lower clearance and increased absorption. When compared to ocular injections, iontophoresis treatment typically has higher patient compliance. It can be mixed with different drug delivery methods. The drawbacks of iontophoresis include its lack of a sustained half-life, the requirement for repeated administrations, minor pain as a side effect in certain circumstances but no risk of infections or ulcers, and possible poor patient compliance due to frequent administrations [151].

Recently, Kim et al. investigated the combination of iontophoresis with nanoparticles. Kim et al. used an iontophoretic technique to deliver latanoprost-loaded PLGA nanoparticles for the treatment of glaucoma. These PLGA nanoparticles offered the benefits of sustained release of latanoprost and extended drug residence time. In vivo, the 300 nm nanoparticles produced the longest-lasting therapeutic effects. This unique method for extending the duration of the drug and decreasing the frequency of drug administration in the treatment of glaucoma is demonstrated by the fact that the drug lasted for more than seven days and exhibited an increase in efficacy by ~23 times when compared to Xalatan^®^, a commercially available latanoprost eye drop [152]. Commercial iontophoresis devices for ocular drug delivery have been developed. One example is OcuPhor/pegaptanib. The OcuPhor^TM^ system is intended for transscleral iontophoresis (DDT) and comes with an applicator, dispersive electrode, and dose controller. In addition, this apparatus transfers the active drug into the retina and choroid. Visulex^TM^, a comparable device, was developed to enable the selective passage of ionized molecules through the sclera [153]. Iontophoresis has also been successfully used to deliver antibiotics, including gentamicin, tobramycin, and ciprofloxacin. However, vancomycin was not a successful candidate due to its high molecular weight. Both antisense oligodeoxyribonucleotides (ODNs) and dexamethasone were delivered successfully. Numerous antibiotics have been effectively injected into the vitreous of rabbit eyes, including gentamicin, cefazolin, ticarcillin, amikacin, and vancomycin. There have also been reports of transscleral iontophoresis with amikacin, gentamicin, steroids (methylprednisolone and dexamethasone), and other drugs [154].

### 4.7. In Situ Gels

**Figure 3:**
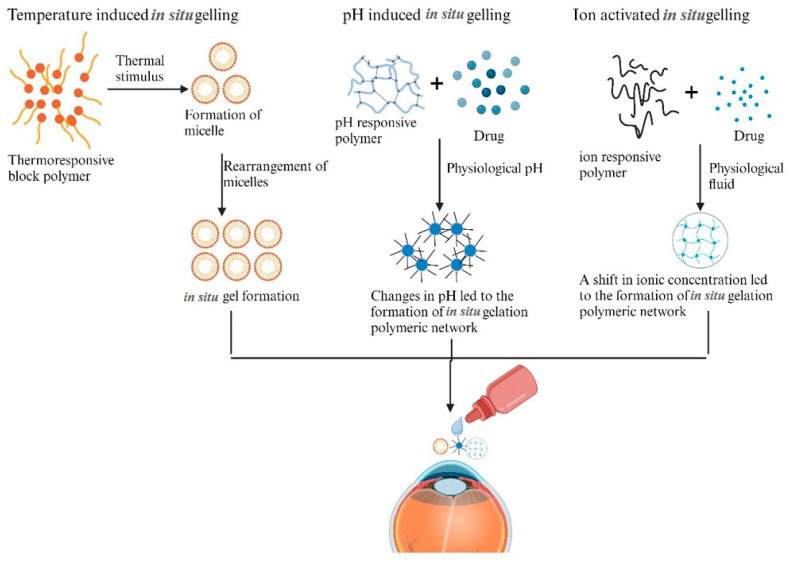
Ophthalmic drug delivery using in situ gelling polymer systems. Created with BioRender.com (accessed on 3 August 2024).

In situ gelling nano-systems are droppable gels that are liquid when injected but undergo a phase transition in the ocular cul-de-sac to form a viscoelastic gel. This transformation can be triggered by various factors including UV radiation, temperature, pH, and ions [155]. The gel-forming polymers used in in situ gels provide a protective shield for the ocular surface, reducing the need for injections and improving patient compliance. This technology has significantly enhanced the distribution of ocular drugs via in situ gel technology [156]. Upon contact with the ocular surface, in situ gelling formulations undergo a phase transformation from a liquid state to a gel-like state. These formulations yield a longer residence time of the drug on the eye, allowing it to stay in close proximity to the tight junctions for a longer duration. The viscoelastic nature of in situ gels facilitates adherence to the cornea, conjunctiva, and surrounding tissues. This prolonged contact facilitates better drug absorption through tight junctions by improving the interaction between the drug-containing gel and the ocular tissues. Furthermore, in situ gels can be prepared in a way that allows the progressive drug to be released over a period of time [157]. The delivery of drugs to the eyes could be revolutionized by in situ gelling, addressing problems such as frequent dosing and poor bioavailability of conventional ophthalmic formulations (e.g., eye drops and ointments). Figure 3 summarizes different in situ gelling systems for ophthalmic drug delivery.

Research in gels has gained recent attention to develop thermosensitive gels that react to temperature changes for ocular administration. There have been reports of a number of thermogelling polymers being used for ocular administration, including chitosan, polycaprolactone, polyethylene glycol, poly (lactide), poly (glycolide), and poloxamers [158]. These thermosensitive polymers combine or pack to create temperature-dependent micellar aggregates, which gel upon an additional temperature increase. These thermosensitive formulations can potentially improve drug bioavailability in relation to the anterior and posterior regions of the eye [159].

Liu et al. investigated gatifloxacin-containing in situ gelling formulations based on alginate/HPMC for ocular administration. Rheological investigations revealed that both the gatifloxacin and blank formulations exhibited gradual shear-thinning, with viscosity decreasing as angular velocity increased. Additionally, after the formulations were diluted with artificial tear fluid, the viscosity increased significantly. When exposed to tear fluid, alginate underwent a transition to a gel phase, whereas 2% HPMC E50Lv solution did not. With 1% alginate and 2% HPMC E50Lv, the optimal formulation demonstrated excellent release properties without causing irritation or tissue injury [160].

Cardoso et al. developed a microemulsion-based in situ gelling formulation for topical administration following eye surgery in order to administer betamethasone and moxifloxacin. The microemulsion possessed properties that guaranteed ocular acceptance. The formulation had an ideal gelling temperature for the ocular surface and was in a liquid condition at storage temperatures, allowing for precise dosing. The microemulsion was found to slow the release of both drugs when the drugs were evaluated in vitro in comparison to controls. An ex vivo study of the microemulsion using porcine cornea with simulated tear fluid showed that the microemulsion had much higher permeability. In situ gelling microemulsion could be a viable alternative for post-intraocular surgery [161].

Shivam et al. developed an in-situ gel filled with moxifloxacin-containing nanoparticles, which showed extended drug release, tolerance in the eyes, and effectiveness against bacterial keratitis. An in vitro diffusion analysis using the Korsmeyer–Peppas model showed a prolonged drug release. Research on the ex vivo efficacy of treating caprine keratitis in infected corneas revealed a decrease in the number of bacteria present. The histology of the corneas showed that the bacterial burden had dropped, and that the corneal epithelium had healed. When applied to the cornea at the prescribed dosage, HET-CAM was tested and showed that the developed formulation was safe and non-irritating. An optimized moxifloxacin-loaded nanoparticle-laden in situ gel would enhance ocular bioavailability while maintaining drug release, antibacterial activity, ocular tolerance, and a transition from a solution to a viscous gel in the eye [162]. Jimenez et al. developed a microsphere/temperature-responsive gel with sustained release qualities to increase the ocular dispersion of cysteamine, a reducing agent used to treat cystine crystals in cystinosis. One drop of the temperature-responsive gel technology based on pNIPAAm released cysteamine during a 12 h period. These findings demonstrated that cysteamine entered the eye after direct application in a well-absorbed form, with most of the drug entering the cornea and minimal levels of the drug entering the bloodstream [163]. Despite improvements, in situ gels face certain challenges. The higher viscosity may impair eyesight and cause pain for the patient, potentially accelerating removal due to reflex blinks and tears. Therefore, to minimize the restrictions to a manageable level, important control of the viscosity should be taken into account throughout the design and optimization of the in situ gel formulation [164].

### 4.8. Contact Lenses

Contact lenses can be hard or soft polymer devices that fit around the cornea to correct refractive defects. These can be made of polymers that are hydrophilic or hydrophobic [165]. Therapeutic contact lenses are a patient-friendly, readily changeable technology that can offer a prolonged release over time [166]. Because of surface tension, they stick to the outer eye’s moist surface once applied. Several drugs have been developed with the intention of being administered via the eyes, including β-blockers, antihistamines, and antimicrobials. The presence of the contact lens permits the drug molecules to remain in contact with the ocular tissue for extended periods of time, with a greater flux passing through the cornea and a reduced amount of the drug being lost via the lacrimal duct [167]. Additionally, when compared to conventional eye drops, which require frequent dosing, contact lenses enable a higher drug bioavailability due to the longer duration of drug residence on the cornea [168]. Immersing contact lenses in a drug solution is a frequently used way to load them with drugs [169]. Drug-eluting contact lenses are solid dosage forms that offer a significant increase in drug bioavailability due to their high capacity for producing close drug contact with the cornea and prolonged drug residence. Thus, drug-loaded contact lenses provide a number of benefits, including a reduction in the total quantity of drug needed, a decrease in the frequency of dosing, and a reduction in the amount of drug lost due to systemic absorption [170]. The first commercial drug-eluting contact lens (ACUVUE^®^ Theravision^®^ with Ketotifen) was released into the market in 2022 to treat the symptoms of ocular allergies. Contact lenses have recently become viable platforms for drug delivery to the anterior segment of the eye [171]. These devices work by first allowing the drug to diffuse in the tear film formed behind the lens, then dispersing it in the tear fluid, and finally allowing it to be absorbed into the cornea. Drug flow from the contact lens to the cornea is prolonged as a result of the drug’s ongoing absorption in the cornea, which keeps the drug concentration in the post-lens tear fluid lower than in the contact lenses. This is known as the sink effect [9]. With this strategy, therapeutic efficacy is increased, drug level variations are reduced, and the necessary dosage is decreased [172]. Furthermore, the use of contact lenses avoids the requirement for preservatives and permeation enhancers commonly found in multidose eye drops, which can cause ocular irritation [173].

Wu et al. evaluated the efficacy of drug delivery in pirfenidone (PFD)-loaded contact lenses made by inserting a PFD and PVA insert between two layers of silicone elastomer. The results showed that the contact lens could increase the residence period of PFD in tears and aqueous humor by five times, whereas the drug load was only one-tenth that of eye drops. Moreover, the PVA addition gives the lens lower protein adsorption properties and increased oxygen permeability. Inhibiting proliferative ocular disorders with a PFD-PVA-loaded contact lens is a promising drug delivery method [174,175]. To cure glaucoma, Ding et al. developed a contact lens device with integrated microtubes. The device can increase the bioavailability of drugs, lower the chance of side effects, and extend the 45-day drug release period. More significantly, the contact lens’s curvature varies in response to variations in intraocular pressure (IOP), which in turn causes increased drug release. This characteristic makes the contact lens an adaptive drug-release device that may offer dynamic and adaptable anti-glaucoma treatment [176]. It is anticipated that contact lenses and nanotechnology will be used in the treatment of ocular illnesses more frequently in the future [176]. In another study, Xu and colleagues developed a micelle–contact lens system that contained latanoprost and timolol. In vivo experiments demonstrated that the contact lens reduced IOP for more than 7 days in the same way that daily commercial eye drops did. Furthermore, the bioavailability of the two drugs on the contact lens was much higher than that of the drugs in the eye drops (measured using collected tear fluid at different time points), indicating that greater amounts of the drugs were available for intraocular absorption [177]. Jiao et al. developed a novel antibacterial and antioxidant contact lens using a polyacrylamide semi-interpenetrating network hydrogel that included quaternary ammonium chitosan and tannic acid. According to the antibacterial test, the contact lens had an approximately 100% bactericidal impact on *Escherichia coli* and *Staphylococcus aureus*. Tannic acid has the additional benefit of reducing oxidative stress and shielding cells from ROS-induced cytotoxicity. Therefore, this drug-free antimicrobial and antioxidant contact lens holds great promise in the management of inflammatory and infectious ocular conditions [178].

### 4.9. Microneedles

**Figure 4:**
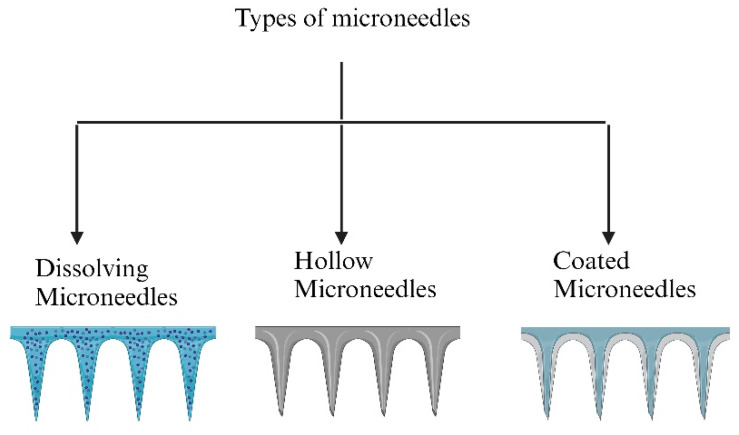
Different types of microneedles for ocular drug delivery. Created with BioRender.com (accessed on 3 August 2024).

Microneedles (MNs) are a promising, minimally invasive approach that offers the benefits of controlled drug release, convenience of administration, and inexpensive manufacturing [179,180]. For vision-threatening posterior ocular disorders like diabetic retinopathy, age-related macular degeneration, and posterior uveitis, this approach may offer an effective treatment regimen. MNs may lessen the possibility of intravitreal injection-related risks and side effects, including bleeding, cataract development, endophthalmitis, and pseudo-endophthalmitis [181,182]. Additionally, therapeutic agents can be delivered to the retina and choroid using an MN drug delivery system. There are various MNs, including solid, hollow, and dissolved MNs, and polymer-coated polymeric MNs have been investigated and tested [183,184] (Figure 4). Microneedles are designed to pierce only the sclera by hundreds of microns, minimizing injury to deeper ocular tissues. Puncturing the sclera and depositing drug solutions or carrier systems can aid in drug diffusion into deeper ocular tissues, choroid, and neuronal retina [185]. Microneedles filled with drugs greatly improved ocular bioavailability as compared to conventional eye drops [186]. Furthermore, microneedles can target medicine delivery to specific tissues, which might decrease drug wastage. Therefore, as a less intrusive method for administering drugs to the eyes, microneedles are being studied worldwide [187,188].

One of the leading causes of blindness on a global scale is fungal keratitis, an infectious corneal disease. Using hyaluronic acid and polylactic acid (PLA), Shi et al. created dissolving microneedle array patches to address this issue. One of these was a 30% PLA–hyaluronic acid MN patch, which reversibly entered the corneal epithelial layer and completely repaired the cornea in 12 h. An investigation carried out using a rabbit model of fungal keratitis showed that the therapeutic impact of self-implantation of drug-loaded MN patches as a controlled release reservoir for local drug delivery is much better than that of eye drops. As a result, the MN patch functions as an effective and quick-healing ocular drug delivery device [189].

An MN-based pen-type device was developed by Song et al. with the goal of improving MN insertion reliability and facilitating facile insertion into a limited target region of ocular tissue. To develop the MN pen, a solid MN made of SU-8 resin was constructed and fastened to a macroscale applicator. The resultant MN was 140 µm in height and 200 × 200 µm in base area. To dip coat the MN, rhodamine B, Evans blue, or sunitinib malate were utilized as model drugs in combination with a polymer carrier. It was demonstrated that the MN pen made it possible to precisely localize drugs within the cornea’s stromal membrane, which is otherwise challenging to accomplish when drugs are applied topically because of the corneal epithelium [190]. In another study, Wu Y. et al. developed a dissolving microneedle patch made of PVA and PVP K29-32. To test the microneedle patch’s ability to transfer proteins to the ocular tissues, they encapsulated ovalbumin in the needles. A silicone mold designed using laser technology held 10 µL of the polymeric blend, and it was discovered that one microneedle array contained 94.52 µg of ovalbumin. During or after the fabrication of the microneedle array, the entrapped ovalbumin did not break down or clump, which was another intriguing finding. This supports the idea that microneedles could preserve the stability of such protein-based materials, which was validated by an SDS-PAGE analysis of ovalbumin recovered from microneedle arrays. Ex vivo studies conducted on porcine sclera revealed that the MNs delivered approximately twice as much ovalbumin as a simple ocular patch or film, partially entered the scleral tissue (at a depth of up to 75% of needle height), and disintegrated within 150 s of insertion. Using Franz diffusion cells, diffusion studies were conducted to determine the quantity of cargo released from the microneedle array. The results showed that within 48 h, over 80% of the cargo was released, surpassing the total amount released by the ocular patch/film. The hen’s egg–chorioallantoic membrane test allowed for the confirmation that the microneedle patch was non-irritating [191].

The microneedle devices described above are less intrusive than other options, such as intravitreal injection, but they carry the risk of infection and inflammation at the injection site. Such complications may lead to additional pain and distress. A prolonged patch application to a patient’s eye will undoubtedly result in discomfort [192]. Currently, there are no microneedle-based ophthalmic formulations available on the pharmaceutical market, except for single microneedle injections. A number of factors, such as the volume of liquid injected, might affect how painful or tolerable the formulation is for the patient. Therefore, every one of these components needs to be thoroughly evaluated and adjusted. Despite the fact that microneedles are made to be as minimally intrusive as possible, any pain can make a patient reluctant or afraid of the procedure [193]. These include the hazards of causing endophthalmitis, pseudoendophthalmitis, and vitreous detachment. Furthermore, the coating procedure reduces the efficiency of the needle’s delivery and sharpness, leading to coated microneedles having poor drug-loading capacity, unstable drug release rates, frequent administration, and low repeatability. Because of these issues, MN therapies for chronic eye illness have not been as successful as other therapies like contact lenses [186].

### 4.10. Hydrogels

Hydrogels are advantageous due to their hydrophilic nature and three-dimensional structure. These systems are able to hold substantial amounts of water, providing several benefits over other methods of ocular drug delivery. Their key advantages include extended drug residence times at the application site, sustained drug release at the intended site, and simultaneous delivery of several drugs [194]. The resilience of hydrogels to flushing and blinking addresses a major barrier to conventional eye drops, which generally maintain ocular contact for a couple of minutes. There are three different ways to administer hydrogels: intravitreal, topical, and intracameral [195]. These systems are effective for delivering drugs due to their high porosity, substantial drug loading capacity, and ability to absorb and release water. Recent advancements have led to the development of more targeted, stimuli-responsive hydrogels, which can release a drug in response to specific stimuli [196]. Diaz-Tome et al. developed a hydrogel formulation using cyclodextrins, hyaluronic acid, carrageenan, and gellan gum to improve the delivery of the antifungal drug econazole. When compared to non-hydrogel solutions, the hydrogel formulations exhibited a controlled release profile, with drug release rates 1.5–3 times slower than solution formulations [197]. To improve drug ocular bioavailability, a thermosensitive glycol chitosan-based levofloxacin hydrogel was developed by Shi et al. The drug-embedded hydrogel had a better C_max_ and AUC_0–12_ h than its aqueous solution. Notably, the hydrogel formulation significantly extended the precorneal retention time of the drug [198].

### 4.11. Bispecific Antibodies

Bispecific antibodies (bsAbs) are genetically engineered molecules that can bind two distinct epitopes on either the same or separate antigens [199]. By targeting both soluble and cell-surface antigens, bsAbs have a broader range of activities compared to monoclonal antibodies (mAbs), thereby offering numerous possibilities for therapeutic applications.

BsAbs can be classified into two main categories based on the presence or absence of the fragment crystallizable (Fc) region [200]. The first group is referred to as IgG-like, since it has an Fc region, and examples include knobs into holes (KiHs), DuoBody, and CrossMab. Given that these bsAbs have a similar structure to full-length IgG, their molecular weight is close to that of mAbs (~150 kDa), they have an extended half-life (t_1/2_), exert effector functions, and exhibit high serum stability [200]. Nevertheless, the administration of these antibodies can be restricted by low tumor tissue penetration, complexity in manufacturing and purification processes, immunogenicity, and adverse effects due to off-target interactions between Fc domains and Fcγ receptors [199].

The second group, known as non-IgG-like or fragment-based subtypes, lacks an Fc region, and examples include single-chain variable fragments (scFv), bispecific T-cell engagers (BiTEs), diabodies, and diabody derivatives. These bsAbs have enhanced permeability and tissue penetration capability due to their smaller size (ranging from 25 to 110 kDa) and reduce the likelihood of non-specific activation of innate immunity [201]. However, they have a short t_1/2_ and poor serum stability. Based on the clinical needs, bsAbs are designed in different formats and can exert different mechanisms of action, including (i) bridging malignant cells and effector cells (T cells, natural killer (NK) cells), (ii) activating or inhibiting multiple receptors, (iii) mimicking a co-factor or an enzyme, and (iv) a piggybacking approach. To gain a comprehensive understanding of the various bsAb formats and mechanisms of action, readers can review the existing literature [199,202,203,204,205,206].

Currently, eleven bsAbs are approved in the US, with most being immune cell engagers for oncology [207]. Following the US FDA’s approval of blinatumomab (Blincyto^®^ by Amgen) in 2014 for B-cell acute lymphoblastic leukemia [208], the therapeutic potential of bsAbs has been investigated for many other indications, including chronic inflammatory, autoimmune, neurological, vascular, ophthalmic, and hematologic disorders and infections. A faricimab bsAb, for example, is indicated for neovascular (wet) AMD, DME, and retinal vein occlusion (RVO).

Faricimab (Vabysmo^®^) acts through the inhibition of two distinct pathways, by neutralizing both angiopoietin-2 (Ang-2) and (VEGF-A). It lowers arterial permeability and inflammation by inhibiting both Ang-2 and VEGF-A, as well as lowering pathological angiogenesis and restoring vascular stability, and has a molecular weight of ~149 kDa [199,209,210]. The product is formulated with a high drug loading (120 mg/mL of faricimab) and is administered as an intravitreal injection of 0.05 mL, equivalent to 6 mg of faricimab. Additionally, it comprises D-sucrose (potentially as a stabilizer), polysorbate 20 (surfactant), L-methionine (antioxidant), L-histidine (buffering agent), sodium chloride (tonicity adjuster), and acetic acid (for pH adjustment to 5.5) as excipients. Its apparent ocular and systemic half-life is ~7.5 days, suggesting a reasonably extended duration of action [210]. Overall, bsAbs offer exciting opportunities for novel drug design and development. With the continued development of modern protein engineering methods and click-chemistry-based designs, they are poised to have a lasting therapeutic impact beyond immune oncology.

### 4.12. Gene Delivery

Gene therapy has gained significant attention from academic communities and medical institutions worldwide over the past three decades. It shows potential as a treatment for glaucoma and other eye diseases [211]. Gene therapy has been investigated for various inherited retinal and optic nerve degenerations, such as X-linked retinoschisis, Usher syndrome, retinitis pigmentosa, choroideremia, Leber congenital amaurosis, achromatopsia, and Leber’s hereditary optic neuropathy, in addition to wet age-related macular degeneration, diabetic macular edema, and retinal vein occlusion [212].

Gene delivery methods utilize various mechanisms to transfer genetic information into host cells, each with unique benefits and drawbacks. Adenoviral and adeno-associated virus (AAV) vectors are two examples of viral vector delivery systems. Adenoviral vectors may carry genes up to 30 kilobases (kb) in length and show good safety and transduction efficiency. However, because of their quick clearance from pre-existing immunity, their application in retinal gene therapy is restricted. There are several non-viral gene delivery techniques, including electroporation, magnetofection, aptamer-based techniques, physical techniques, and gene guns. Physical delivery involves injecting bare plasmid DNA, siRNA, mRNA, or miRNA, but absorption is limited due to rapid degradation [213].

**Figure 5:**
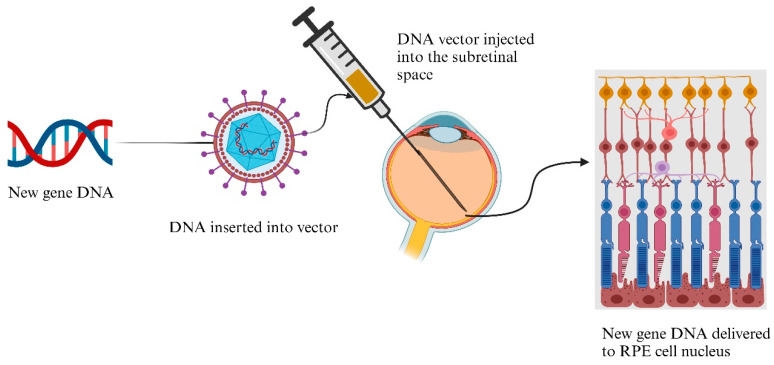
A schematic representation of subretinal gene therapy. Created with BioRender.com (accessed on 3 August 2024).

Luxturna^TM^ (voretygene neparvovec), approved by the US FDA in 2017, is the first viral ocular gene therapy to treat Leber’s congenital amaurosis type 2 [214]. It introduces the gene encoding the isomerohydrolase enzyme using an AAV vector in patients with a biallelic mutation of the *Rpe65* gene [215,216]. It can be administered to the eyes intravitreally, subretinally, or suprachoroidally. The subretinal (Figure 5) and suprachoroidal approaches need vitrectomy or specialized suprachoroidal delivery devices, and although they offer the advantage of delivering the vector close to the intended target, they are logistically challenging. The use of suprachoroidal delivery, which is comparable to intravitreal delivery in terms of complexity, is already being used clinically to treat disorders like uveitis, as evidenced by the use of Xipere (triamcinolone acetonide) [217].

SYL040012 is a novel siRNA drug candidate that selectively blocks the β2 adrenergic receptor in the ciliary body to lower high IOP in glaucoma and ocular hypertension. The in vivo silencing effectiveness was evaluated using normotensive New Zealand White rabbits, resulting in a 20% IOP reduction that lasted for up to 190 h [218]. For viral infections like viral keratitis, mRNA-based CRISPR technology can distribute Cas9 mRNA and target the vial genome at the same time. Yin et al. used mRNA-CRISPR/Cas9 technology to show that intrastromal delivery of HSV-1-erasing lentiviral particles can directly target the HSV-1 genome. By removing the virus from the trigeminal ganglion reservoir in various study models, they were able to effectively block HSV-1 replication and occurrence. Notably, no significant off-target effects were found in the complete genome sequence [219]. Jain et al. reported that glaucoma phenotypes in in vitro, in vivo, and ex vivo models were restored by targeted editing of the mutant MYOC gene (Y437H mutation) using CRISPR/Cas9. Targeting the first exon of MYOC, the CRISPR/Cas9 system was inserted into adenovirus 5 and administered intravitreally to the desired location in order to completely eradicate the expression of mutant MYOC in mice models. The mutant MYOC in TM cells was effectively shut down by the CRISPR/Cas9 system, which also lessened ER stress brought on by the misfolded protein buildup. Furthermore, CRISPR/Cas9 targeted MYOC knockdown resulted in a reduction in IOP in the glaucomatous mouse model. Using an ex vivo human organ culture paradigm, this was the first publication to successfully demonstrate the use of CRISPR/Cas9 for human genome editing, with encouraging findings in the knockdown of endogenous MYOC [220]. Additionally, injection of gene medicines can suppress VEGF, a crucial stimulator of neovascularization in wet AMD. This inhibits the creation of new blood vessels and slows the reduction in symptoms. AGN211745 (previously Sirna-027) is a chemically modified naked siRNA. In order to treat wet macular lesions, the target gene, VEGFR-1, induces gene silencing by binding complementary target RNA with lytic cytoplasmic protein complexes known as RNA-induced silencing complexes. This significantly reduces the level of VEGFR-1 mRNA [221]. Shen et al. used choroidal CNV mice and retinal mice models to study Sirna-027 delivery. They found that both IVT and periocular injection of Sirna-027 resulted in detectable levels in the retina for 4 to 5 days, suggesting prolonged retention in retinal cells and a consequent decrease in VEGFR-1 mRNA and protein [222].

One of the drawbacks of gene delivery is the difficulty in delivering the therapeutic gene to the specific cells in the retina. Though there are ongoing attempts to develop and refine intraocular delivery routes, there is not yet an ideal delivery mechanism that can consistently and efficiently reach the target cells in every patient [223]. Another possibility is that the immune system may mistake the viral vector for an invader, resulting in a heightened level of cellular and humoral immunological responses [224]. Depending on the amount and mode of distribution, gene-therapy-associated inflammation may manifest as anterior, intermediate, or posterior uveitis. The degree of ocular inflammation can be influenced by the type of transgene, promoter, and viral vector. Compared to AAVs, adenovirus vectors are linked to higher degrees of inflammation [225]. Lastly, the practicality of providing gene therapy on a bigger scale is uncertain due to the high prices and complicated procedures involved. This raises important ethical questions regarding the accessibility and availability of treatment [226].

A summary of different ocular drug delivery systems along with the routes of administration, advantages, and limitations is presented in Supplementary Table S1.

## 5. Pharmaceutical Technologies for Ocular Applications

### 5.1. Three-Dimensional Printing Technologies

Conventional approaches like eye drops and oral drugs have shown promise in treatment, but poor patient compliance, limited bioavailability, and daily treatment burden with eye drops underscore the need for sustained implanted drug delivery devices. Additionally, eye drop dosages are limited due to the eye cul-de-sac’s capacity (30 µL) [227]. This has led to the development of several ophthalmic formulations, including those for use in contact lenses, intraocular insertions, lacrimal plugs, and resorbable conjunctival formulations. There have been some investigations into the three-dimensional (3D) printing of ophthalmological devices; however, understanding and research into ophthalmic formulations using 3D printing technology are still in its early stages.

**Figure 6:**
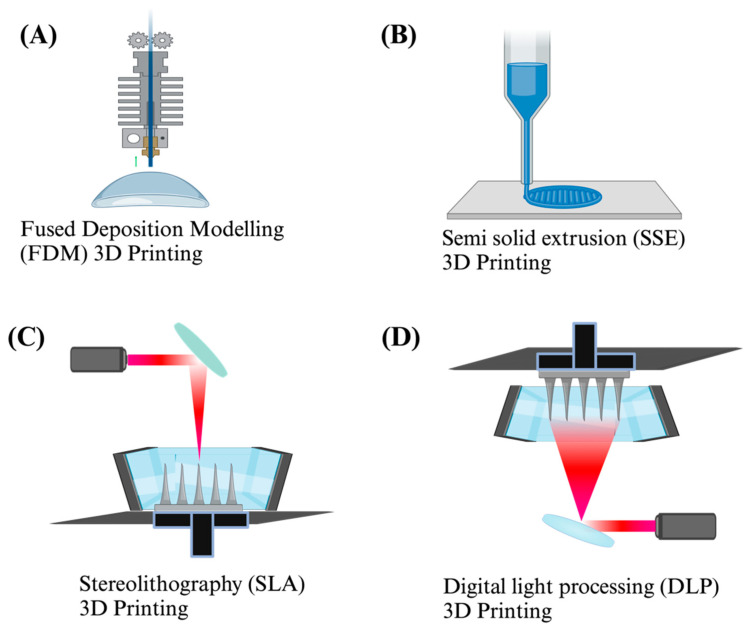
Schematic diagram of different types of 3D printing (3DP) technologies: (**A**) fused deposition modeling (FDM), (**B**) semi-solid extrusion (SSE), (**C**) stereolithography (SLA), and (**D**) digital light processing (DLP). Created with BioRender.com (accessed on 27 August 2024).

Three-dimensional printing, also broadly referred to as additive manufacturing (AM), is the process of combining materials, often in a layer-by-layer fashion, to construct objects of varying shapes and sizes based on computer-aided design (CAD) models or digital scans (Figure 6) [228,229]. Due to its compact yet versatile and automated features, this technology has been utilized to fabricate customized pharmaceutical dosage forms and medical devices that are specifically designed to meet the unique demands of each patient. This improves the effectiveness of treatment and the overall results for patients [230]. Additionally, its unique portable nature also enables it to administer therapies directly at the point of care, such as in hospital settings or community pharmacies or in remote locations, for example, even in space exploration. Furthermore, due to its capability to develop low-cost individual items, each with its own distinct shape and precision, it can be utilized for small-scale manufacturing in preclinical and clinical testing, where there is a requirement for frequent adjustment of dosage based on the PK-PD profile [231,232]. It is, therefore, noteworthy to explore this transformative technology and its application for pharmaceutical ocular applications.

#### 5.1.1. Fused Deposition Modeling

Among different 3D printing technologies, the material extrusion (MM) 3D printing technique has been most commonly utilized for developing 3D-printed ocular devices or API delivery platforms. Depending on the types of feedstocks used in the extrusion process, MM is further categorized into fused deposition modeling (FDM), direct powder extrusion (DPE), and semi-solid extrusion (SSE) or bioprinting [229]. Briefly, FDM uses filament composed of thermoplastic polymers and APIs which is melted and extruded via a nozzle onto a build platform, using a driver motor. This process allows for the creation of a complex product based on the CAD model [233]. Though FDM can generate complex objects with microscale resolution, additional steps such as support removal, cleaning, and polishing or finishing are required to enhance the visual appearance of the printed items. FDM offers several advantages, including the flexibility of using a large variety of polymeric feedstock materials that are regarded by the FDA as Generally Regarded as Safe (GRAS), cheap costs for the printer and maintenance, high mechanical properties of the finished product, and fast production time [234]. Additionally, varying concentrations of APIs can be loaded into the polymeric filament either by hot-melt extrusion (HME) or by immersing a ready-made filament into an API solution, allowing for passive diffusion into the filament. Nevertheless, each of these methods has its own set of constraints. HME coupled with FDM is mostly preferred. However, the APIs and polymers should withstand high temperatures during the HME process, which restricts its application for thermolabile drugs. On the other hand, passive diffusion can only achieve minimal drug loading, which restricts its use for high-drug-load products [235].

The use of topical medications for glaucoma treatment and management is limited by factors such as tear turnover, blinking, induced lacrimation, tear film, and solution drainage. Traditional hypodermic treatments are not preferred due to the need for specialized healthcare personnel, discomfort, and low patient compliance. For such a scenario, Mohamdeen et al. and colleagues developed personalized ocular contact lenses loaded with timolol maleate (TML) using 3DP technology for glaucoma [236]. They used a single-screw HME coupled with FDM 3D printing technique to produce lenses that initially appeared yellowish due to drug melting. However, when printed below the drug’s melting point at 200 °C, the lenses became transparent. The drug release was prolonged for 7 days but faced challenges like burst release due to localized TML, low bioavailability due to drug permeation, and corneal irritation. Wu et al. investigated the influence of FDM 3D printing nozzle and printer stage temperatures to develop microneedle arrays (MNs) using polylactic acid (PLA) filaments and then coated them with rhodamine B (model drug) [237]. The printing stage was heated above the glass transition temperature (Tg) of the PLA (60–120 °C), while the nozzle was set below 210 °C to prevent decomposition of the PLA. They found that increasing thermal parameters during the FDM process improved interfacial bonding strength and decreased the void density of PLA layers.

#### 5.1.2. Semi-Solid Extrusion

FDM 3D printing uses filaments (generally 1.75 mm diameter) as feedstock material, while SSE 3D printing uses gel or paste layers to fabricate desired objects. It has found applications in drug delivery, bioprinting, and tissue engineering due to its low printing temperatures, single-step processing, and single-use disposable needles or prefilled cartridges, which can aid in fulfilling regulatory standards for quality [234,238]. Additionally, it is a single-step process, eliminating filament fabrication and enhancing efficiency and product consistency. As biocompatible materials and bio-inks for SSE 3D printing become more available, researchers are exploring the potential of this technique for ocular therapies [239].

Tagami et al. developed controlled-release levofloxacin-loaded ocular patches using the SSE technique for corneal antibacterial application. These patches were printed using hydrogel-based inks containing mannitol, xylitone, and HPMC and then lyophilized [240]. Mucoadhesive hydrophilic polymers undergo hydration and swelling, resulting in the formation of a viscous hydrogel. Such a hydrogel aids in preventing drug drainage and facilitates prolonged drug release [51]. Loannou et al. developed a 3D-printed implant containing 5-fluorouracil (5-FU) to prevent conjunctival fibrosis after glaucoma surgery [241]. The implant, made of polycaprolactone (PCL) and chitosan (CS), was fabricated using a heat extrusion technique and loaded with 1% 5-FU. The implant showed desired biocompatibility and biodegradability, with sustained drug release behavior over 8 weeks, possibly obviating the need for repetitive 5-FU injections in glaucoma patients in clinical settings. In another group, Paleel and colleagues used the SSE 3D printing approach to develop intracameral implants with high drug loads (5 and 10%) of timolol for the treatment of glaucoma [242]. The intracameral implant showed a sustained-release profile, releasing the drug over 8 weeks without significant cytotoxicity to human trabecular cells. These case studies demonstrate the potential of 3D printing for developing biocompatible and personalized ocular implantable sustained drug release in treating glaucoma, potentially enhancing adherence to ocular medication and improving therapy results.

#### 5.1.3. Vat Photopolymerization

While FDM and SSE are frequently studied in pharmaceutical manufacturing, their use is limited when printing ophthalmic products due to their intricate shapes and small sizes due to their larger nozzle size (>1 mm diameter) and time-consuming post-processing treatments such as the removal of supports and surface polishing. In such instances, vat photopolymerization (VPP)-based 3D printing methods, including digital light processing (DLP) and stereolithography (SLA), are favored due to their ability to provide higher resolution with a small layer thickness and a smooth surface finish [234,243]. These techniques create solid objects by photopolymerizing a vat of liquid resin (polymerizing monomers) via light irradiation, in a layer-by-layer fashion. SLA printers employ UV light to selectively cure liquid resin layers, while DLP utilizes a digital projector to simultaneously expose the entire polymer resin to UV light, thereby achieving a quicker processing time than SLA printers [244]. The printing process occurs at room temperature, avoiding the thermal degradation of drugs and making it suitable for thermolabile drugs and even for large molecules.

**Figure 7:**
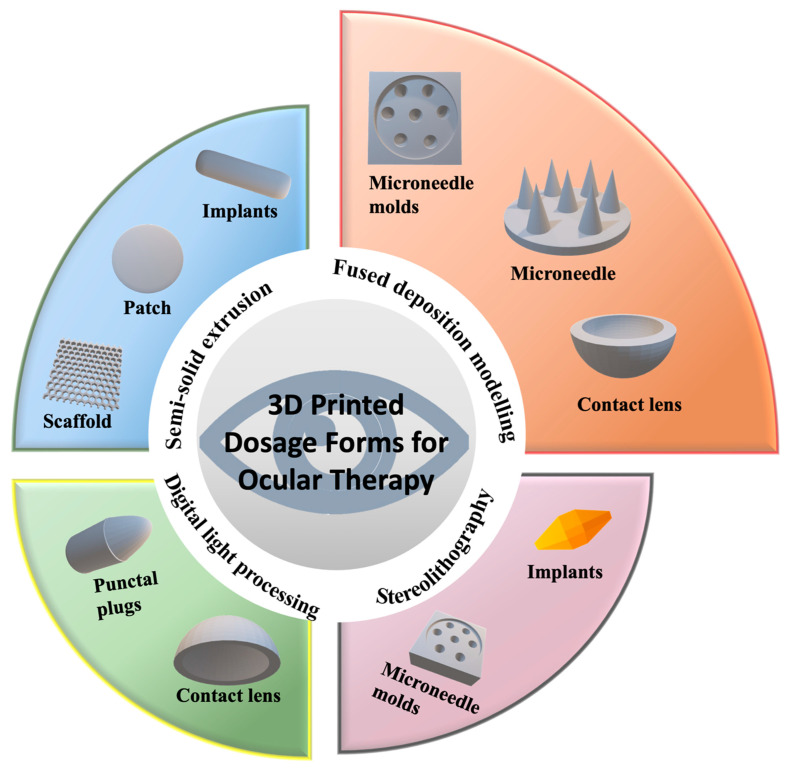
Various ocular dosage forms fabricated using different 3D printing technologies.

SLA 3D printing has been used to develop molds for the fabrication of MNs. For example, Fitaihi et al. investigated key parameters of the self-administrable MN design for optimum ocular tissue penetration [245]. The SLA 3D-printed cone-shaped MNs, with a 1:2 aspect ratio, demonstrated superior accuracy and effectiveness. Researchers also highlighted the importance of fine-tuning printing parameters like layer thickness and orientation angle to achieve desired MN characteristics. Another research group showcased the use of SLA 3D printing to create 3D molds, which were then used to produce placebo (without API) dissolving MNs using HPMC and PVP K90 [246]. Both in SLA and DLP 3D printing, polyethylene glycol diacrylate (PEGDA) has been the most commonly used crosslinking monomer agent due to its high biocompatibility. Xu et al. utilized DLP 3D printing to create punctal plugs loaded with dexamethasone [247]. Plugs containing a mixture of 20% *w*/*w* PEG 400 and 80% *w*/*w* PEGDA exhibited continuous release for 7 days, while plugs made entirely of PEGDA showed extended release for over 21 days. This suggests that DLP 3D printing is a promising 3D printing method for creating individualized drug-loaded plugs for treating dry eye symptoms. Goto et al. developed azithromycin-loaded contact lenses using the DLP 3D printing technique with PEGDA and PEG 400, a photoinitiator [240]. The lenses showed sustained drug release profiles (30–40% release in 1–2 h), and changing the ratio PEGDA to PEG 400 resulted in different release rates. The weight of the lenses increased by 20–30% post-dissolution, with high crosslinking and high PEGDA concentration reducing the increase in weight. The lenses also showed antimicrobial effects with an inhibition zone diameter of 30 mm, comparable to commercial eye drops. In another study, researchers developed contact lenses using DLP-based 3D printing, using clear resin monomer [248]. The materials had a tensile modulus and Young’s modulus of 5.56 ± 0.13 MPa and 3.78 ± 0.08 MPa, respectively. The 3D-printed lenses were modified with a nanostructure pattern using laser ablation, which can serve as a transducer for detecting ocular parameters. These case studies demonstrate DLP 3D printing’s potential for creating customized drug-loaded ophthalmic devices and integrating optical biosensors in smart wearable diagnostics. However, the main limitation of VPP 3D printing is the limited availability of photocrosslinkable polymers and their absence on the FDA’s GRAS list. Figure 7 demonstrates different 3D technologies that have been applied to prepare various ophthalmic dosage forms.

Over the past decade, 3D printing technology has garnered tremendous traction, particularly in personalized medicine for retinal and corneal conditions; however, it faces numerous challenges. Some of these factors include limited excipients, software and instrument limitations, finished product mechanical properties such as strength, surface roughness, and compliance with regulatory requirements. In ocular formulation or delivery device development, SSE and VPP technologies are mostly preferred; nevertheless, residual solvents in many final 3D-printed formulations remain a major limitation. In addition, the restricted use of crosslinking photopolymerizing chemicals has limited the applicability of these technologies [234]. It is imperative to conduct extensive research in order to create excipients that are sustainable, biocompatible, biodegradable, and non-toxic for the 3D printing of ocular products. Currently, most 3D printers do not meet GMP requirements, which may render their products unsafe for human consumption. Additionally, the high start-up capital costs with 3D printers and materials and cost advantages due to economies of scale also hinder entry into healthcare systems. In-line or off-line non-destructive characterization techniques, for example, process analytical technologies (PATs), can be integrated instead of destructive methods. Furthermore, quality control measurements should be in place to ensure policies imposed by regulatory bodies are met. Overall, future advancements in software and hardware, improvements in printing process factors, faster printing speed, the combination of medical scanners with printers, and stringent quality control measures could further increase 3D printing’s accessibility in ocular therapy. Table 2 summarizes the recent research on ophthalmic formulation preparation using different 3D printing technologies.

### 5.2. Hot-Melt Extrusion

**Figure 8:**
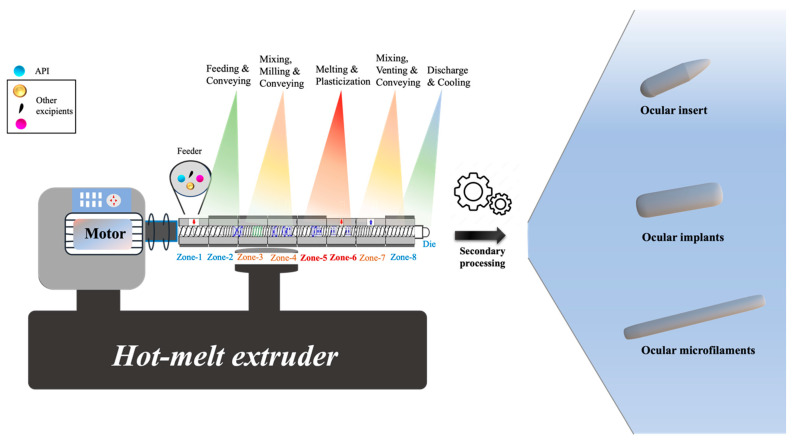
A schematic representation of critical hot-melt extrusion steps and ocular dosage forms manufactured with HME technology.

HME is a widely used processing technology in pharmaceutical manufacturing that originated from the plastic industry in the mid-19th century. HME consists of five main stages (Figure 8): material input, high-temperature melting and plasticization, mixing and conveying, discharging, and cooling [253]. The drug(s) and excipient(s) are introduced into the extruder feeder and then subjected to either co-rotating or counter-rotating intermeshing screws at temperatures typically higher than the glass transition temperature (Tg) of polymers, and often even higher than the melting temperature (Tm). It enables effective dispersive and distributive mixing of materials, while resulting in molecular-level mixing between an API and more or more excipient, leading to extrudates with high content uniformity and improved quality. Subsequently, the extrudate is discharged via a die aperture, cooled, and collected [254].

HME has emerged as a viable alternative to traditional methods for the manufacture of solid-oral, ocular, topical, and other tailored novel formulations. Mechanical shear stress and thermal energy can disrupt the crystalline lattice structure of a crystalline drug/polymer, resulting in amorphous, co-amorphous, and co-crystal formulations that can be utilized to increase the aqueous solubility and bioavailability of poorly water-soluble APIs. These formulations have diverse uses, including modified-release, targeted-release, abuse-deterrent, and taste-masking uses. The notable advantages of HME technology include continuous manufacturing; being an economical, solventless method; and its industrial-scale feasibility, automation potentiality, high reproducibility, and ability to be monitored in real time. Additionally, the extruder screw elements, such as conveying, kneading, and mixing components, can be customized according on the specific materials being processed and the required level of mixing intensity for the desired application. Thus, customized screw arrangements can be used to process delicate APIs/excipients, making HME very versatile and adaptable to diverse industries. Furthermore, HME can be integrated with additional pieces of equipment, such as numerous feeders, or processes, such as 3D printing, to create bespoke ocular inserts, implants, and other products. PAT instruments, including as UV-VIS and NIR, are increasingly used in HME lines to monitor crucial process parameters such as API content uniformity, extrudate size, residence time distribution (RTD), degradation, amorphous content, and many more. For example, proteins, peptides, and other heat-sensitive biologics are not ideal candidates for HME. In-depth pre-formulation information is helpful for the rational selection of API(s) and excipients(s) for the HME process to fabricate desired products. Table 3 lists the recent case studies of hot-melt-extruded dosage forms for ophthalmic formulation preparation.

### 5.3. Injection Molding

Injection molding is a widely used manufacturing technique known for its ability to produce complex shapes with precision and efficiency in the medical device industry [262]. Injection molding has become particularly relevant for creating implantable devices that provide sustained drug release [263]. This method allows for the fabrication of biodegradable or non-biodegradable polymeric implants [262,263]. Formulation considerations through injection molding require a balance of biocompatibility, drug stability, and release kinetics [264]. Polymers such as poly(lactic-co-glycolic acid) (PLGA) are often used for this manufacturing process [265]. The choice of polymer, excipients, and drug loading methods must be made in consideration of the specific ocular environment, which has unique pH, enzymatic activity, and clearance mechanisms [266]. This process offers several advantages, including scalability and reproducibility. However, challenges arise in ensuring drug stability, especially for thermolabile drugs [265,266]. The selection of process parameters, such as temperature, pressure, and cooling rates, must be optimized to prevent drug degradation or changes in polymer structure that could affect drug release profiles. Moreover, the small size and intricate design of ocular implants pose technical difficulties in achieving precise and uniform drug loading [267]. Table 4 lists the advantages and the limitations of manufacturing ocular dosage forms using emerging technologies, including 3DP, HME and injection molding.

## 6. Role of Digital Healthcare, AI, and ML

The primary stage where digital tools can be applied for healthcare would be the application of analyzing medical images, such as retinal and ocular scans, and predicting the minute changes that occur in the ocular tissue to detect early signs of diseases like diabetic retinopathy or macular degeneration [268]. For instance, Google’s DeepMind has developed an algorithm that can detect over 50 different eye diseases with an accuracy that matches the diagnosis by an ophthalmologist [269]. This application would help cover a larger chunk of the world’s population with affordable healthcare. Automated AI-based screening tools can be implemented in primary care or remote settings, allowing non-specialists to perform preliminary checks [270]. This can significantly reduce the burden on ophthalmologists and ensure that patients at risk are referred for further examination promptly [271,272]. This approach can be looped into monitoring the disease progression using wearable devices and disease monitoring. Devices like smart contact lenses can continuously monitor intraocular pressure (IOP) in glaucoma patients. These devices can alert patients and healthcare providers to changes that might indicate a worsening of the condition, allowing for timely intervention [273].

These tools can be used to process large amounts of data right from early-stage to late-stage pharmaceutical development. AI can be used to process large amounts of biochemical and pharmacological data for target identification for varied pathophysiologies [274]. This information can also be used in turn for discovering new candidates to target these discovered sites. Machine learning models can predict which compounds are most likely to be effective against specific molecular targets involved in ocular diseases, thus streamlining the early stages of drug discovery [275,276,277].

Current drug therapies for these diseases, involving multiple daily eye drops or frequent injections, are effective but can be challenging to maintain and tolerate in the long run [278]. This has spurred scientific efforts to create delivery systems that can attach to eye cell components and extend the therapeutic effects of the medications they deliver safely. In 2023, the US FDA approved an implant (iDose TR) for reducing IOP for patients with open-angle glaucoma and ocular hypertension, which releases drugs directly into the eye [279]. Although this device provided longer-lasting effects compared to drops or injections, extended use sometimes led to eye cell death, necessitating a return to traditional treatments like eye drops and injections.

AI and ML have also found their applications in the field of drug delivery and product development. Researchers at Johns Hopkins Medicine’s Wilmer Eye Institute and the University of Maryland recently showed that artificial intelligence models and machine-learning algorithms can be used to successfully predict components of amino acids that make up therapeutic proteins are most likely to safely deliver therapeutic drugs to animal eye cells [280].

Hsueh et al. developed a machine-learning-based approach to design multifunctional peptides for ocular drug delivery. These peptides are engineered to bind effectively to components of eye cells, enhancing the duration and effectiveness of drug delivery. The study demonstrated the potential of these engineered peptides to improve the sustainability and efficiency of ocular treatments, potentially reducing the frequency of eye drop applications and injections required for managing ocular diseases. This developed model was validated their through extensive experimentation and analysis, highlighting its promise in advancing ocular drug delivery systems [281].

By integrating digital healthcare with AI and ML, the management of ocular diseases can be revolutionized. Early and accurate detection, personalized treatment plans, and continuous monitoring can significantly improve patient outcomes. In the pharmaceutical and drug delivery industry, these technologies can accelerate drug development, optimize delivery methods, and enhance patient compliance, ultimately leading to better management of chronic ocular conditions and improved quality of life for patients.

The patents listed (refer to the Supplementary Table S2) highlight a variety of innovative approaches aimed at overcoming the inherent challenges associated with ocular drug delivery, such as limited bioavailability, patient compliance, and precise targeting of therapeutic agents. Many of the patents focus on developing sustained-release drug delivery systems to enhance therapeutic outcomes by maintaining consistent drug levels over extended periods. Examples include Ocular Therapeutix’s sustained-release ophthalmic composition (US11020235B2) and Alcon’s ocular insert for sustained drug delivery (US11305007B2). These technologies aim to reduce the frequency of administration, thus improving patient compliance. Another trend seen has been the use of nanoparticles for drug delivery, as seen in patents by Mati Therapeutics (US11090292B2) and Novartis AG (US11065334B2). Clearside Biomedical’s patents (US10537546B2 and US11505538B2) focus on microinjectors and microneedles for targeted delivery. These systems are emerging as a minimally invasive method for precise drug administration. Advancements in technology and integration of engineering principles and tools to formulation and drug delivery sciences are exemplified by Google’s smart contact lens (US11789046B2), which combines drug delivery with health monitoring capabilities. This trend points towards a future where smart devices could play a significant role in personalized medicine and real-time health management.

Certain trends can be observed based on the clinical trials ongoing (refer to Supplementary Table S3), the patents filed, and the products introduced in the market. One such trend is the increased focus on use of biocompatible and biodegradable excipients to enhance patient safety and comfort while ensuring effective drug release. The integration of advancing technologies in the pharmaceuticals and devices suggests a future where ocular drug delivery systems may also include diagnostic capabilities, enabling real-time monitoring and tailored treatment regimens. Future innovations are expected to prioritize patient convenience and adherence. Systems that require less frequent dosing, are minimally invasive, and improve user comfort will be at the forefront of ocular drug delivery research and development. The growing number of patents and the emphasis on sustained release and targeted delivery systems indicate a strong potential for these innovations to achieve regulatory approval and commercial success. Companies are likely to invest in technologies that demonstrate clear clinical benefits and patient adherence advantages.

In summary, the patents granted since 2020 reflect a dynamic and rapidly evolving field of ocular drug delivery, with significant advancements in sustained release systems, nanoparticle-based delivery, microneedle technologies, hydrogel and biodegradable implants, and smart devices. These innovations aim to improve therapeutic outcomes, enhance patient compliance, and provide more targeted and personalized treatments for various ocular conditions.

## 7. Industry and Regulatory Perspectives

Ophthalmic formulations (for topical or local drug delivery) are defined by the US FDA as one type of complex product [282], which is more challenging in chemistry, manufacturing, and control (or CMC) during the drug development of these products as compared to conventional ones. In the past decades, the US FDA has initiated research grants/contracts in collaboration with prestigious academic laboratories and a few contract research organizations globally to understand advanced scientific and technical solutions to solve challenging regulatory science problems for complex generic products. With these efforts, funding has been awarded to investigate different ophthalmic formulations such as ointments, emulsions, and implants [283,284]. The knowledge gained through these grants has helped to advance the knowledge (through publications), provide scientific rationale for abbreviated new drug applications (ANDAs) or NDAs, facilitate new or updated regulatory guidance, and accelerate complex generic approvals. A new general draft guidance for industry, “*Quality Considerations for Topical Ophthalmic Drug Products*”, has been released in 2023 [285]. In addition, numerous product-specific guidance (PSG) statements have been issued or revised to provide detailed recommendations for the development and evaluation of specific ophthalmic drug products. It is highly advantageous to consult the general guidance and PSGs in order to stay informed about the latest recommendations from the agency regarding ophthalmic drug product development.

While significant advancements have been made in many areas, a persistent challenge remains in developing robust in vitro–in vivo correlations (IVIVCs) for ophthalmic drug products. The first IVIVC guidance from the US FDA issued in 1997 is for extended-release oral dosage forms [286], serving as the cornerstone for subsequent adaptations for other dosage forms. Although the US FDA does not mandate the development of IVIVCs for submission processes, it is essential for the industry to have a better understanding of IVIVC establishment when feasible. In general, Level A IVIVC (point to point) is the most informative and recommended by the US FDA. Developing Level A IVIVC is difficult for conventional dosage forms such as oral products, and it is even more challenging for complex ophthalmic products. Considerations need to be made in three aspects: (1) bio-predictability of the developed in vitro release testing method; (2) relevancy of pharmacokinetic (PK) profiles of the drug (in different segments of the eye) following administration; and (3) methodologies (e.g., conventional numerical deconvolution, mechanical models such as physiological-based PK) used for IVIVC establishment. In addition, the determination of drug concentration in different chambers or tissues of the human eye is often infeasible. Despite the risk of translation from animals to humans, it is worthwhile to examine whether a preclinical IVIVC can be developed using an animal model such as rabbits.

## 8. Conclusions

It is imperative to facilitate ophthalmic drug products to address the unmet medical needs associated with eye disorders. This review provides a roadmap for the development of ophthalmic drug products, emphasizing a more product-oriented approach. In addition, this review encompasses crucial insights into physiological barriers, treatment strategies, eye disorders, and drug delivery systems (excipients and drug substances). The knowledge serves as a valuable foundation for the development of an efficient delivery strategy capable of precisely delivering a drug to the target site within the desired duration. In addition, it underscores the significance of understanding advanced and emerging technologies that can be applied to manufacture ophthalmic drug products, such as 3D printing and hot-melt extrusion. Lastly, it is important to adhere to regulatory recommendations, including general guidance and product-specific guidance, to help better develop ophthalmic drug products.

## Figures and Tables

**Table 1:** Ophthalmic implants approved by US FDA (including discontinued ones) [108].

Active Ingredient	Proprietary Name	Strength	Indication	Route of Administration	Efficacy Duration	Rate Controlling Systems	Year of Approval
Ganciclovir	Vitrasert	4.5 mg	Cytomegalovirus	Implantation	5–8 months	PVA and EVA	1996
Fluocinolone acetonide	Retisert	0.59 mg	Uveitis	Intravitreal	30 months	PVA	2005
Dexamethasone	Ozurdex	0.7 mg	DME, RVO, and uveitis	Intravitreal	Up to 6 months Depends on the conditions	PLGA	2009
Fluocinolone acetonide	Iluvien	0.19 mg	DME	Intravitreal	36 months	Polyimide tube, PVA, and silicone adhesive	2014
Fluocinolone acetonide	Yutiq	0.18 mg	Uveitis	Intravitreal	36 months	Polyimide tube, PVA, and silicone adhesive	2018
Bimatoprost	Durysta	10 mcg	Reducing IOP	Ophthalmic/intracameral	Several months	PLGA, PDLA, PDLLA and PEG3350	2020
Ranibizumab	Suvismo	2 mg	Wet AMD	Intravitreal	Up to 6 months	Port delivery system	2021
Travoprost	iDose TR	75 mcg	Reducing IOP	Intracameral	3 months	Titanium reservoir coated with semipermeable membrane	2023

**Table 2:** Summary of recent studies on ophthalmic formulation preparation using different 3D printing technologies.

Device/Dosage Forms	APIs (Drug Load % *w*/*w*)	Excipients	3D Printing Technologies	Findings/Applications	Ref.
Contact lenses	Timolol maleate (1%)	EVA and PLA	HME-FDM	Drug-eluting contact lenses provided an initial burst release followed by a sustained release for 3 days for the treatment of glaucoma.	[236]
Microneedles (MNs)	Rhodamine B (model drug)	PLA	FDM	The MN can be coated with APIs for various biomedical uses.	[237]
Molds for dissolving MNs	Galantamine hydrobromide	PLA filaments, PVA/PVP	FDM	FDM 3D printing was used to develop molds which were used to prepare API-loaded MNs	[249]
Ophthalmic patches	Levofloxacin (0.5%)	HPMC, mannitol, and xylitol	SSE	Antibacterial effect for eye infections. Most of the drug is released within 60–120 min.	[240]
Intracameral implants	Timolol maleate (5–10%)	PCL	SSE	The 3D-printed implants were developed to deliver sustained drug release over eight weeks for treating glaucoma.	[242]
Implants	5-fluorouracil (1%)	PCL and chitosan	SSE	The implant was developed to prevent conjunctival fibrosis post-glaucoma surgery.	[241]
Hydrogel-based scaffold	Betamethasone sodium phosphate (2.5%)	Polyethyleneimine	SSE	The 3D-bioprinted hydrogel scaffold has the potential to manage ocular inflammation.	[250]
Dissolving MNs	Placebo	PVP and PVA	SLA	Placebo MNs developed for potential ocular applications.	[245]
Molds for dissolving MNs	Placebo	PLA	SLA	The molds were 3D-printed to produce HPMC and PVP K90 dissolving MNs.	[246]
Punctal plugs	Dexamethasone (10–20%)	PEGDA and PEG 400	DLP	Plugs made with 100% PEGDA showed prolonged releases for over 21 days for treating dry eye disease.	[247]
Contact lenses	Azithromycin (1%)	PEGDA and PEG 400	DLP	The lenses demonstrated antimicrobial properties with an inhibition zone diameter of 30 mm, similar to commercial eye drops.	[251]
Hollow MNs	Placebo	Biocompatible commercial resins	DLP	An angled-printed MN showed optimal geometries compared to a flat-printed (at 0° to the base plate) MN.	[252]

HME (hot-melt extrusion), FDM (fused deposition modeling), SSE (semi-solid material), DLP (digital light processing), SLA (stereolithography), EVA (ethylene-vinyl acetate), PLA (polylactic acid), HPMC (hydroxypropyl methylcellulose), PCL (polycaprolactone), PEGDA (polyethylene glycol diacrylate), PEG 400 (polyethylene glycol 400), polyvinyl pyrrolidone (PVP), PVA (polyvinyl alcohol).

**Table 3:** Summary of recent studies on the preparation of ophthalmic formulations using hot-melt extrusion technology.

Dosage Form	APIs	Excipients	Key Findings	Refs.
Fixed-dose combination ocular inserts	Prednisolone sodium phosphate and Sulfacetamide sodium	PEO, HPC-HF, and EC	The HPC-HF- and EC-containing inserts showed sustained drug release profiles and were stable for >90 days at 25 and 40 °C. Optimum bio-adhesive strength and smooth surface finish were observed, making them suitable for topical ocular application.	[255]
Inserts	Valacyclovir HCL	HPC EF-HPMC K4M and PEG 400	The ocular inserts were fabricated to treat corneal keratitis. The inserts showed a sustained drug release profile, dissolving completely in 8 h, and enhanced permeation.	[256]
Inserts	Brinzolamide (BRZ)	HPMC and Poloxamer 407	The solubility and residence duration of BRZ in the polymer matrix were influenced by various interactions, including ionic, Van der Waals, H-bonding, and electrostatic forces. The inserts showed a sustained-release profile for 24 h and better IOP control and remained stable at ambient temperature and 4 °C for 6 months. Drug release was governed by swelling, polymer chain relaxation, and diffusion phenomena.	[257]
Biodegradable implant	Dexamethasone	PLGA	The implant showed an irregular surface with 6% internal porosity and a triphasic drug release profile. Physicochemical characterizations revealed limited interaction between the drug and the polymer, resulting in a two-phase system of dexamethasone crystals embedded within a PLGA matrix. The reverse-engineered implant and Ozurdex showed similar compositions and structural similarities, allowing for an equivalent in vitro release profile.	[258]
Biodegradable intracameral implant	Prostamide	PEG 3350 and PLGA	The implant is rod-shaped and formed by a hot-melt extrusion process, with the implant being 150 to 300 μm in diameter or width, 0.50 to 2.5 mm in length, and 30 to 100 μg in total weight. It effectively reduces IOP for at >2 months after placement in the anterior chamber of the eye.	[259]
Monofilamnt (500–700 μm)	Diclofenac potassium	PEG–PCL–chitosan–keratin blend	Amorphous and miscible solid dispersions were created. Rapid and sustained drug release rates were achieved with the PEG/PCL/chitosan/keratin blends at various combinations. Presence of hydrophilic and phobic polymers improved the solubility of the diclofenac potassium with a tunable release rate.	[260]
Monofilamnt (20 μm)	Carvdilol	Eudragit^®^ E	Up to 20% of carvedilol was loaded. Fast release of carvedilol, which has poor water solubility. Comparable drug loading and drug release with suture fibers with similar compositions produced by solvent-free melt electrospinning and solvent-based electrospinning.	[261]

PEO (polyethylene oxide), HPC (hydroxypropyl cellulose), EC (ethyl cellulose).

**Table 4:** Advantages and limitations of manufacturing ocular dosage forms using 3D printing (3DP), hot-melt extrusion (HME) and injection molding techniques.

Pharmaceutical Technologies	Advantages	Limitations
3D printing	Customizable dosage forms tailored to specific patient needs can be designed and printed. Highly flexible and able to manufacture complex and small-sized geometries. Small footprint Capable of low-cost production of individualized products, which is especially useful for preclinical and clinical testing. 3DP can be integrated with other processes like HME to produce complex ocular products for sustained drug release.	Lack of regulatory and quality control guidelines on 3D-printed dosage forms. Limited material choices; for example, thermally unstable drugs/polymers are unfit for FDM. Batch manufacturing processes and slower print speeds limit it for industrial-scale production. Often requires post-processing of the final 3D-printed dosage forms. Mostly limited to “proof-of-concept” to this date.
Hot-melt extrusion	Industrial-scale, green, and reproducible process. Continuous process and quality control can be maintained using in-line or off-line PAT tools. High and consistent drug loading can be achieved. Capable of enhancing the solubility of poorly water-soluble drugs.	Limited to thermally stable drugs/polymers. Often requires post-processing steps such as sizing of final dosage forms. High processing temperature makes it unfit for large molecules such as proteins and peptides. Requires specialized equipment, high start-up costs, and expertise.
Injection molding	Highly precise fabrication method. Scalable and reproducible. Easy to monitor with in-line quality control tools such as PAT.	High temperature may not be suitable for a large number of small molecules and biologics. Limited by small ocular space requiring miniaturization. Difficulty in achieving consistent drug loading throughout the dosage form. Requires specialized equipment and expertise.

## Supplementary Materials

The following supporting information can be downloaded at https://www.mdpi.com/article/10.3390/pharmaceutics16101325/s1: Table S1: Summary of different ocular drug delivery systems with their route of administration, advantages, and limitations; Table S2: List of Patents demonstrating advancements in ocular drug delivery (2020–2024); Table S3: List of clinical trials carried out by industrial sponsors currently in phase 3 and 4 studying formulation and drug actions (2020–2024).
