# Supplementary material for: Advancements in Ocular Therapy: A Review of Emerging Drug Delivery Approaches and Pharmaceutical Technologies
Source: Pharmaceutics. 2024 Oct 12;16(10):1325. doi: 10.3390/pharmaceutics16101325 (PMC11511072; doi:10.3390/pharmaceutics16101325)
Supplement: Supplementary file 1 [file pharmaceutics-16-01325-s001.zip › pharmaceutics-3207990-supplementary.pdf]

Supplementary Table S1. Summary of different ocular drug delivery systems with their route of administration, advantages, and limitations

S. No.	Ocular delivery systems	Route of administration	Advantages	Limitations
1	Implants	Intravitreal or subconjunctival	• Offer a sustained and controlled release of drugs for the long term.<br>• Targeted delivery.<br>• Reduces the frequent drug administration.<br>• Effective in treating posterior eye conditions.<br>• Provides localized delivery.	• Challenges in the development and commercialization of biodegradable implants for macromolecules for eye disorders.<br>• Requires surgical procedures.<br>• Lack of adaptability: once implanted, it could be difficult to change the drug or the dosage.<br>• Some ocular implants could irritate or cause discomfort over time.<br>• Risk of infection.
2	Liposomes	Topical or intravitreal injection	• Beneficial for large molecular weight drugs.<br>• Both hydrophilic and hydrophobic drugs can be encapsulated in liposomes to increase their stability and solubility in ocular formulations.	• Short residence time.<br>• Liposomes exhibit physical and chemical instability.<br>• Liposomes have a limited capacity to load drugs, particularly hydrophilic drugs.
3	Nanoparticles	Topical, Intravitreal, or subconjunctival injection	• These are smaller and less irritating to the eye.<br>• Provides continuous drug release to minimize repeat doses<br>• Facilitates greater absorption and enhances intracellular penetration.<br>• Targeted distribution to desired tissues.	• Difficulty in achieving homogeneous particle dispersion.<br>• Insufficient drug loading.<br>• Harmful effects are caused by the type and dose of surfactants used.<br>• When applied topically, nanoparticles frequently experience quick clearance in the eye.<br>• Physical stability of nanoparticles can be an issue during storage. It may require extreme low temperature control (*e.g.*, -80°C).
4	Nanomicelles	Topical or intravitreal injection	• Nanomicelles can considerably increase the solubility of poorly water-soluble drugs.<br>• These are well tolerated by the eye because of their biocompatibility.<br>• Improves permeability across ocular barriers.	• The stability of nano micelles is challenging.<br>• Limited drug loading.<br>• Drugs can be released continuously using nano micelles, although exact control over the release rate is challenging to achieve.

5	Microparticles	Subconjunctival, Intravitreal, or periocular injection	• Drugs that are unstable or poorly soluble can have their bioavailability increased by encapsulating them in microparticles.<br>• Microparticles can be designed to deliver different drugs at the same time, allowing for combination therapy with synergistic effects in treating complicated eye diseases.<br>• Microparticles can protect sensitive drugs from degradation.	• Microparticles frequently encounter difficulties in reaching the deeper ocular tissues, particularly the posterior section of the eye.<br>• Microparticles may have burst release which leads to dose dumping.<br>• It can be difficult to provide consistent dosage with microparticles, particularly when exact drug quantities are required for successful treatment outcomes.<br>• Sterilization of microparticle system may be challenging.
6	Iontophoresis	Trans-scleral, Trans-corneal	• Iontophoresis offers localized drug delivery to the target ocular tissue.<br>• Drugs delivered using iontophoresis can attain higher concentrations in ocular tissues than topical formulations.<br>• Iontophoresis enables precise control of drug delivery by altering the intensity and duration of the electric current.	• Sensitive eye tissues may be damaged by the electrical current used in iontophoresis, particularly if it is not well managed.<br>• This method is only suitable for small molecules.<br>• This may cause irritation or discomfort in people.<br>• Iontophoresis devices are relatively sophisticated and pricey, making them less accessible in some clinical settings.<br>• It requires repeated administration, which might damage the ocular surface.
7	*In situ* gels	Topical	• Sustained drug delivery, reducing the frequency of administration.<br>• The increased retention time and close contact with the cornea increase drug absorption, which results in higher bioavailability.	• Insufficient drug penetration.<br>• *In situ* gels remain susceptible to quick removal by blinking and tear secretion.<br>• Gel physical stability.<br>• Insufficient drug loading.<br>• Controlling the rate of drug release can be challenging, particularly if the gel degrades or dissolves unevenly.

8	Contact Lens	Topical	• Sustained drug release.<br>• Targeted delivery.<br>• Easy to wear for better patient compliance.<br>• Reduced tear dilution.<br>• Protection of sensitive drugs.<br>• Potential for combination therapy.	• It is difficult to control drug release rates precisely.<br>• Limited drug loading capacity.<br>• Contact lenses are effective in delivering drugs to the anterior segment but less successful in treating disorders in the posterior portion of the eye.<br>• Improper handling of contact lenses, especially when loaded with drugs, increases the possibility of microbial contamination and illness.
9	Microneedles	Trans-scleral, Trans-corneal	• Drugs can be delivered locally and directly to the ocular tissues.<br>• Offers enhanced drug penetration.<br>• Microneedles can deliver a wide variety of therapeutic drugs, such as small molecules, biologics etc.	• For microneedles to be dissolvable or biodegradable, the materials utilized must be biocompatible without producing toxicity. If residual material remains in the eye due to incomplete disintegration or degradation, it may irritate the eye.<br>• It is still difficult to scale up the production of microneedle devices for industry usage.<br>• Limited drug loading.
10	Hydrogels	Subconjunctival, intravitreal injection, or Topical	• Non-invasive administration.<br>• Hydrogels are suitable for administering all kinds of drugs.<br>• Sustained drug delivery at the intended site.	• Hydrogels can easily be removed by blinking and tears.<br>• Achieving exact control over drug release rates can be difficult.<br>• Hydrogels have the potential to temporarily blur vision, particularly if they remain on the cornea for a long period of time.<br>• Limited drug loading.<br>• Hydrogels can degrade in specific environmental conditions.

11	Bispecific antibodies	Intravitreal injection	• Compared to conventional treatments, bispecific antibodies may provide the opportunity for longer dose intervals.<br>• When treating chronic illnesses that require long-term care, bispecific antibodies can reduce the development of resistance by targeting numerous disease pathways at once.<br>• Bispecific antibodies can attach to two separate antigens or epitopes at once, resulting in a more effective and comprehensive therapeutic response.	• Due to their size, these have limited tissue distribution in the eye, particularly in the deeper regions such as the retina.<br>• It is more difficult to manufacture and scale up. It can be difficult to ensure purity and consistency during manufacturing.<br>• These are costly to produce because of the complex nature of their design and manufacturing.
12	Gene delivery	Intravitreal, subretinal injection, or suprachoroidal	• One of the most significant advantages of gene therapy is its ability to give long-term or even permanent treatment by modifying the genetic material in eye cells.<br>• Gene delivery addresses the fundamental cause of genetic eye problems by fixing faulty genes.<br>• Gene delivery can provide new therapy options for diseases that presently lack viable medicines.	• Difficult in delivering the therapeutic gene to the specific cells in the retina.<br>• Reaching the posterior region of the eye, particularly the retina, is challenging.<br>• There is no ideal delivery mechanism that can consistently and efficiently reach the target cells.<br>• Risk of the immune response or inflammation in the eye.<br>• Numerous vectors can only transport a certain amount of genetic material.

Supplementary Table S2. List of Patents demonstrating advancements in ocular drug delivery (2020-2024).

Patent Number	Title	Assignee	Description	Date Granted
US11925578B2	Ocular Implant for Controlled Drug Delivery	Glaukos Corp	An implant designed for controlled drug release within the eye, featuring an outer shell with drug barriers and specific membranes for targeted drug delivery.	April 23, 2024

US10537546B2	Methods and Compositions for Treating Ocular Diseases	Kala Pharmaceuticals	Involves the use of mucin secretagogue compounds to enhance ocular drug delivery by increasing mucus production, thereby improving drug retention.	February 4, 2020
US10851224B2	Ocular Drug Delivery Devices and Methods	Clearside Biomedical	Focuses on a microinjector device for delivering drugs to the suprachoroidal space, enhancing drug dispersion and efficacy.	December 1, 2020
EP3719276A1	Local Ocular Delivery of KIO-100 Family Compounds	Kiora Pharmaceuticals	Covers small molecules inhibiting the enzyme DHODH to reduce inflammatory effects in the retina for treating autoimmune diseases.	August 23, 2023
WO2020163871 A1	Controlled Release Compositions for Ocular Drug Delivery	University of California	A formulation for controlled release of ocular drugs, utilizing biodegradable polymers to achieve sustained drug delivery.	August 13, 2020
US11311472B2	Ocular Drug Delivery System	Clearside Biomedical, Inc.	A system for delivering drugs to the eye, particularly targeting the suprachoroidal space.	Apr 26, 2022
US11090292B2	Nanoparticle Ocular Drug Delivery System	Mati Therapeutics Inc.	Utilizes nanoparticles for enhanced drug delivery to ocular tissues.	Aug 17, 2021

US11389765B2	Sustained Release Ocular Drug Delivery Device	Alcon Inc.	A device for the sustained release of drugs to the eye, improving therapeutic outcomes.	Jul 26, 2022
US10926038B2	Biodegradable Ocular Drug Delivery Implant	Allergan, Inc.	Implantable device that biodegrades after delivering the drug.	Feb 23, 2021
US11167087B2	Injectable Ocular Drug Delivery System	Genentech, Inc.	System for delivering drugs via injection to specific parts of the eye.	Nov 9, 2021
US11351543B2	Ocular Drug Delivery Using Microneedles	MicroOptx, Inc.	Utilizes microneedles for precise delivery of drugs to the eye.	May 17, 2022
US11602549B2	Drug Delivery via Contact Lenses	Johnson & Johnson Vision Care, Inc.	Contact lenses designed to deliver therapeutic agents to the eye.	Mar 7, 2023
US11376312B2	Lipid-Based Nanocarriers for Ocular Drug Delivery	Eyepoint Pharmaceuticals, Inc.	Lipid-based nanocarriers for improved drug delivery and retention in ocular tissues.	Aug 30, 2022
US11534378B2	Ocular Insert for Drug Delivery	Ocular Therapeutix, Inc.	An insertable device that provides controlled drug release to the eye.	Nov 15, 2022
US11509871B2	Advanced Topical Ocular Drug Delivery System	Santen Pharmaceutical Co., Ltd.	Enhanced topical formulation for better bioavailability and therapeutic effect.	Nov 29, 2022

CN112190542B	Aqueous in-situ gel ophthalmic preparation for treating xerophthalmia	Aiwei Pharmaceutical Zhuhai Co. Ltd.	An aqueous in-situ gel ophthalmic preparation used to treat dry eye condition.	Jan 28, 2022

Note: All patent information was retrieved from publicly accessible databases, including the Google Patent Search and the United States Patent and Trademark Office (USPTO) website, accessed on Aug 27, 2024.

Supplementary Table S3. List of clinical trials carried out by industrial sponsors currently in phase 3 and 4 studying formulation and drug actions (2020-2024)

Trial identifier	Title	Indications	Interventions	Sponsor	Phase
NCT04357795	Effect of Cequa in Subjects with Dry Eye Disease	Dry Eye Disease	DRUG: CequaTM (Cyclosporine 0.09%) ophthalmic solution	Sun Pharmaceutical Industries Limited	4
NCT06248619	A Trial to Investigate Teprotumumab Subcutaneous Administration Compared with Placebo in Male and Female Adult Participants With Moderate-to-severe Active Thyroid Eye Disease	Thyroid Eye Disease	BIOLOGICAL: Teprotumumab |OTHER: Placebo	Amgen	3

NCT06106828	A Study To Evaluate The Efficacy, Safety, Pharmacokinetics, And Pharmacodynamics Of Satralizumab In Participants With Thyroid Eye Disease (SatraGO-2)	Thyroid Eye Disease	DRUG: Satralizumab|OTHER: Placebo	Hoffmann-La Roche	3
NCT05723770	Effects of NOV03 on the Tear Film	Dry Eye Disease (DED)	DRUG: instillation of NOV03	Bausch & Lomb Incorporated	4
NCT05401357	Bioequivalence Study With Clinical Endpoint Comparing Bimatoprost Ophthalmic Solution 0.01% and LUMIGANÂ® in the Treatment of Chronic Open-Angle Glaucoma or Ocular Hypertension in Both Eyes.	Glaucoma, Open-Angle|Ocular Hypertension	DRUG: Test - Bimatoprost 0.01% Ophthalmic Solution|DRUG: Reference - LUMIGANÂ® (Bimatoprost 0.01% Ophthalmic Solution)	Amneal Pharmaceuticals, LLC	3
NCT04285580	A Phase 3b Study Evaluating the 24-Hour Intraocular Pressure Lowering Effect of Bimatoprost SR in Patients With	Open-Angle Glaucoma|Ocular Hypertension	DRUG: Bimatoprost SR|DRUG: LUMIGAN	AbbVie	3

	Open-Angle Glaucoma or Ocular Hypertension				
NCT05346354	Efficacy and Safety Study of Ravulizumab IV in Pediatric Participants With NMOSD	Neuromyelitis Optica Spectrum Disorder	DRUG: Ravulizumab	Alexion Pharmaceuticals, Inc.	3
NCT04786574	A Study to See if Tolvaptan Can Delay Dialysis in Infants and Children Who at Enrollment Are 28 Days to Less Than 12 Weeks Old With Autosomal Recessive Polycystic Kidney Disease (ARPKD)	Autosomal Recessive Polycystic Kidney Disease (ARPKD)	DRUG: Tolvaptan (OPC-41061)	Otsuka Pharmaceutical Development & Commercialization, Inc.	3
NCT06131437	A Research Study to See How Well CagriSema Compared to Tirzepatide Helps People With Obesity Lose Weight	Obesity	DRUG: Cagrilintide|DRUG: Semaglutide|DRUG: Tirzepatide	Novo Nordisk A/S	3
NCT06122662	AMX0035 and Progressive Supranuclear Palsy	Progressive Supranuclear Palsy|PSP|Neurodegenerative Diseases|At	DRUG: AMX0035|OTHER: Placebo	Amylyx Pharmaceuticals Inc.	3

		ypical Parkinsonism			
NCT05608291	A Trial to See if the Combination of Fianlimab With Cemiplimab Works Better Than Pembrolizumab for Preventing or Delaying Melanoma From Coming Back After it Has Been Removed With Surgery	Melanoma	DRUG: Fianlimab|DRUG: Cemiplimab|DRUG: Pembrolizumab|DRUG: Placebo	Regeneron Pharmaceuticals	3
NCT05511038	A Study to Learn How Safe the Study Drug Intravitreal (Given by an Injection Into the Eye) Aflibercept is in Participants in India With Diabetic Macular Edema	Diabetic Macular Edema	DRUG: Aflibercept (Eylea, VEGF Trap-Eye, BAY86-5321)	Bayer	4
NCT05666804	Study Assessing the Efficacy and Safety of a Personalized Monotherapy Regimen of Brolucizumab in Patients With Symptomatic Macular	Macular Polypoidal Choroidal Vasculopathy (PCV)	DRUG: Brolucizumab 6mg|DRUG: Brolucizumab 6mg	Novartis Pharmaceuticals	3

	Polypoidal Choroidal Vasculopathy				
NCT05946941	A Study to Evaluate the Efficacy and Safety of Deucravacitinib in Adults With Active SjÃ¶gren's Syndrome	SjÃ¶gren's Syndrome	DRUG: Deucravacitinib|OTHER: Placebo	Bristol-Myers Squibb	3
NCT05926583	A Study of AAV5-hRKp.RPGR for the Treatment of Japanese Participants With X-linked Retinitis Pigmentosa	X-Linked Retinitis Pigmentosa	GENETIC: AAV5-hRKp.RPGR|GENETIC: AAV5-hRKp.RPGR	Janssen Pharmaceutical K.K.	3
NCT06182358	Impact of XDEMVYÂ® on Demodex Blepharitis in Soft Contact Lens Wearers	Demodex Blepharitis	DRUG: Lotilaner ophthalmic solution, 0.25%|DRUG: Vehicle control	Tarsus Pharmaceuticals, Inc.	4
NCT04837482	A Study to Assess the Impact and Adverse Events of Topical Eyedrops of AGN-190584 on Night-driving Performance in Participants, 40 to 55 Years of Age	Presbyopia	DRUG: AGN-190584	Allergan	3
NCT05282004	Study of the Safety of Use of Intravitreal	Neovascular Age-related Macular	DRUG: SOK583A1	Sandoz	3

	SOK583A1 Provided in a Vial Kit	Degeneration (nAMD)			
NCT04853251	A Study Assessing Corneal Endothelial Cells in Patients With Neovascular Age-related Macular Degeneration Treated With the Port Delivery System With Ranibizumab (PDS)	Neovascular Age-related Macular Degeneration	DEVICE: SUSVIMO Port Delivery System with ranibizumab (PDS)|DRUG: LUCENTIS (ranibizumab injection)	Genentech, Inc.	4
NCT04674800	Extension Study of MYL-1701P-3001 for Safety and Efficacy	Diabetic Macular Edema	BIOLOGICAL: MYL-1701P, a proposed biosimilar to Eylea	Mylan Pharmaceuticals Inc	3
NCT03097315	Suprachoroidal Injection of CLS-TA in Patients With Non-infectious Uveitis (AZALEA)	Uveitis	Drug: 4 mg CLS-TA Suprachoriodal Injection	Clearside Biomedical Inc	3

Note: All clinical trial data was retrieved from ClinicalTrials.gov, accessed on August 27, 2024.

References

1. U.S. Food and Drug Administration. Prescribing information: Lifitegrast
2. U.S. Food and Drug Administration. Prescribing information: Rhopressa
3. U.S. Food and Drug Administration. Prescribing information: Vyzylta
4. U.S. Food and Drug Administration. Prescribing information: Luxturna
5. U.S. Food and Drug Administration. Prescribing information: Oxervate
6. U.S. Food and Drug Administration. Prescribing information: Beovu

7. U.S. Food and Drug Administration. Prescribing information: Rocklatan
8. U.S. Food and Drug Administration. Prescribing Information: Vabysmo
9. U.S. Food and Drug Administration. Prescribing information: Izervay
10. U.S. Food and Drug Administration. Prescribing information: Xdemvy
11. U.S. Food and Drug Administration. Prescribing information: Miebo
